# Genetic basis of I-complex plasmid stability and conjugation

**DOI:** 10.1371/journal.pgen.1010773

**Published:** 2023-06-22

**Authors:** Zheng Jie Lian, Minh-Duy Phan, Steven J. Hancock, Nguyen Thi Khanh Nhu, David L. Paterson, Mark A. Schembri

**Affiliations:** 1 Institute for Molecular Bioscience (IMB), The University of Queensland, Brisbane, Queensland, Australia; 2 School of Chemistry and Molecular Biosciences, The University of Queensland, Brisbane, Queensland, Australia; 3 Australian Infectious Diseases Research Centre, The University of Queensland, Brisbane, Queensland, Australia; 4 The University of Queensland Centre for Clinical Research, Brisbane, Australia; Uppsala University, SWEDEN

## Abstract

Plasmids are major drivers of increasing antibiotic resistance, necessitating an urgent need to understand their biology. Here we describe a detailed dissection of the molecular components controlling the genetics of I-complex plasmids, a group of antibiotic resistance plasmids found frequently in pathogenic *Escherichia coli* and other *Enterobacteriaceae* that cause significant human disease. We show these plasmids cluster into four distinct subgroups, with the prototype IncI1 plasmid R64 subgroup displaying low nucleotide sequence conservation to other I-complex plasmids. Using pMS7163B, an I-complex plasmid distantly related to R64, we performed a high-resolution transposon-based genetic screen and defined genes involved in replication, stability, and conjugative transfer. We identified the replicon and a partitioning system as essential for replication/stability. Genes required for conjugation included the type IV secretion system, relaxosome, and several uncharacterised genes located in the pMS7163B leading transfer region that exhibited an upstream strand-specific transposon insertion bias. The overexpression of these genes severely impacted host cell growth or reduced fitness during mixed competitive growth, demonstrating that their expression must be controlled to avoid deleterious impacts. These genes were present in >80% of all I-complex plasmids and broadly conserved across multiple plasmid incompatibility groups, implicating an important role in plasmid dissemination.

## Author summary

Antimicrobial resistance is one of the greatest threats to human health. Left unchecked, we risk a rapid escalation of untreatable infections. Plasmids are one of the most important vehicles for resistance gene carriage and transmission between bacteria, and thus an understanding of plasmid biology is crucial to controlling the spread antimicrobial resistance. Here, we combine advanced bioinformatics and a state-of-the-art genetic screen to understand the molecular mechanisms involved in the maintenance and spread of a group of plasmids strongly associated with antibiotic resistance among bacteria that cause human infection. We characterised genes involved in the replication and maintenance of these plasmids, and experimentally demonstrated that plasmid spread is dependent on a well-conserved secretion system. Our genetic screen also discovered a set of broadly conserved, uncharacterised genes that adversely impact host fitness and plasmid spread under dysregulation. Taken together, these findings describe a molecular blueprint for the biology of a group of clinically relevant antibiotic resistance plasmids found frequently in Gram-negative bacterial pathogens.

## Introduction

Plasmids are extra-chromosomal double-stranded DNA molecules that contribute significantly to the global antibiotic resistance crisis by facilitating the horizontal dissemination of resistance genes via conjugation [1]. The I-complex plasmids, originally grouped together due to morphological and serological similarities in their pili [2], consist of the incompatibility (Inc) groups IncB/O, IncK1, IncK2, IncI1, IncI2, IncIɣ, and IncZ. The replication of these plasmids depends on a replicon structure conserved across the I-complex comprising (i) *repA*, which encodes a replication initiation protein that binds to the downstream origin of replication (*oriV*); (ii) *repB*, a leader peptide sequence upstream of *repA* whose translation is necessary for *repA* expression; and (iii) a small antisense RNA (RNAI) upstream of *repB* which inhibits the translation of *repB* and controls RepA expression, plasmid replication, and plasmid copy number [3–5]. The RNAI sequence also mediates incompatibility between members of the I-complex within the same cell by acting *in trans* [6]. Apart from the phylogenetically distant IncI2 plasmids which do not have a detectable RNAI homolog [7, 8], I-complex plasmids are typed based on similarity to an amplicon upstream of *repA* (encompassing approximately half of the RNAI) using the widely adopted *in silico* replicon typing tool PlasmidFinder [9]. Four phylogenetically distinct clades of I-complex plasmids have been characterised based on *repA* phylogeny: IncI2, IncK1/IncIɣ, IncI1/IncB/O, and IncK2/IncZ [8].

Nearly all I-complex plasmids examined carry antibiotic resistance genes, and these plasmids have been found in *Escherichia coli* strains of commensal [10, 11], clinical [12–14] and animal [11, 15] origin, as well as other *Enterobacteriaceae* [16, 17]. Despite this, knowledge surrounding the conjugation of I-complex plasmids has been mostly derived from the IncI1 plasmid R64. Plasmid R64 contains the following sets of genes involved in conjugation: *tra* genes which encode the I-class mating pair formation (MPF_I_) type IV secretion system (T4SS); *pil* genes which encode the type IV pilus (distinct from T4SS) responsible for cell to cell contact in liquid mating; *nikAB* which encode the P-family mobility system (MOB_P_); *traABCD* which encode regulators of transfer gene expression; and *trbABC* which encode for proteins involved in general conjugative functions [16, 18]. In I-complex plasmids, surface conjugation (conjugation on a surface of solid medium such as agar or filter paper) involves the process of plasmid DNA transfer from donor to recipient cell and requires genes in the *tra* region, *traBC*, *trbAC*, and the *nikAB* genes [19–21]. Liquid conjugation (conjugation in liquid media), in addition to requiring the same genes as in surface conjugation, requires prior establishment of cell-cell contact mediated by genes in the *pil* region [2, 22, 23]. These genetic requirements for conjugation have been extrapolated from R64 to other I-complex plasmids despite extensive amino acid sequence variation in many of the conjugation proteins [11, 15, 24, 25]. Thus, studies identifying the genetic requirements for plasmid replication/maintenance and surface conjugation of I-complex plasmids with conjugation regions distantly related to R64 are lacking.

Transposon-directed insertion site sequencing (TraDIS) is a high-throughput genome-wide screening methodology that enables the simultaneous identification of all genes that play a functional role under a defined condition of interest [26]. Briefly, a highly saturated transposon mutant library is subjected to selection under a condition of interest, and differences in transposon insertions between pre- and post-selection libraries can be used to determine the importance of every gene under the condition tested. This methodology has been used as a genetic screen to identify bacterial genes involved in complex phenotypes, for example in *E*. *coli* where it has been used to define genes involved in resistance to human serum [27], zinc [28], and polymyxins [29], as well as the production of cell surface factors [13, 30].

Here, we employed TraDIS to define genes required for the replication/stability and conjugation of a poorly characterised subgroup of I-complex plasmids represented by the plasmid pMS7163B, a completely sequenced and conjugative IncB/O/K/Z plasmid isolated from an *E*. *coli* pyelonephritis strain [13]. Our screen also identified a strand-specific transposon insertion bias upstream of several previously uncharacterised genes. These genes were located in the early transfer region of pMS7163B and their overexpression adversely impacted host cell fitness. We posit these genes belong to a newly identified category of highly conserved genes whose expression must be controlled to avoid deleterious repercussions, suggesting they play an important role in the biology of I-complex plasmids in *E*. *coli* and other pathogenic *Enterobacteriaceae*.

## Results

### I-complex plasmids can be classified into two major clusters

**Fig 1:**
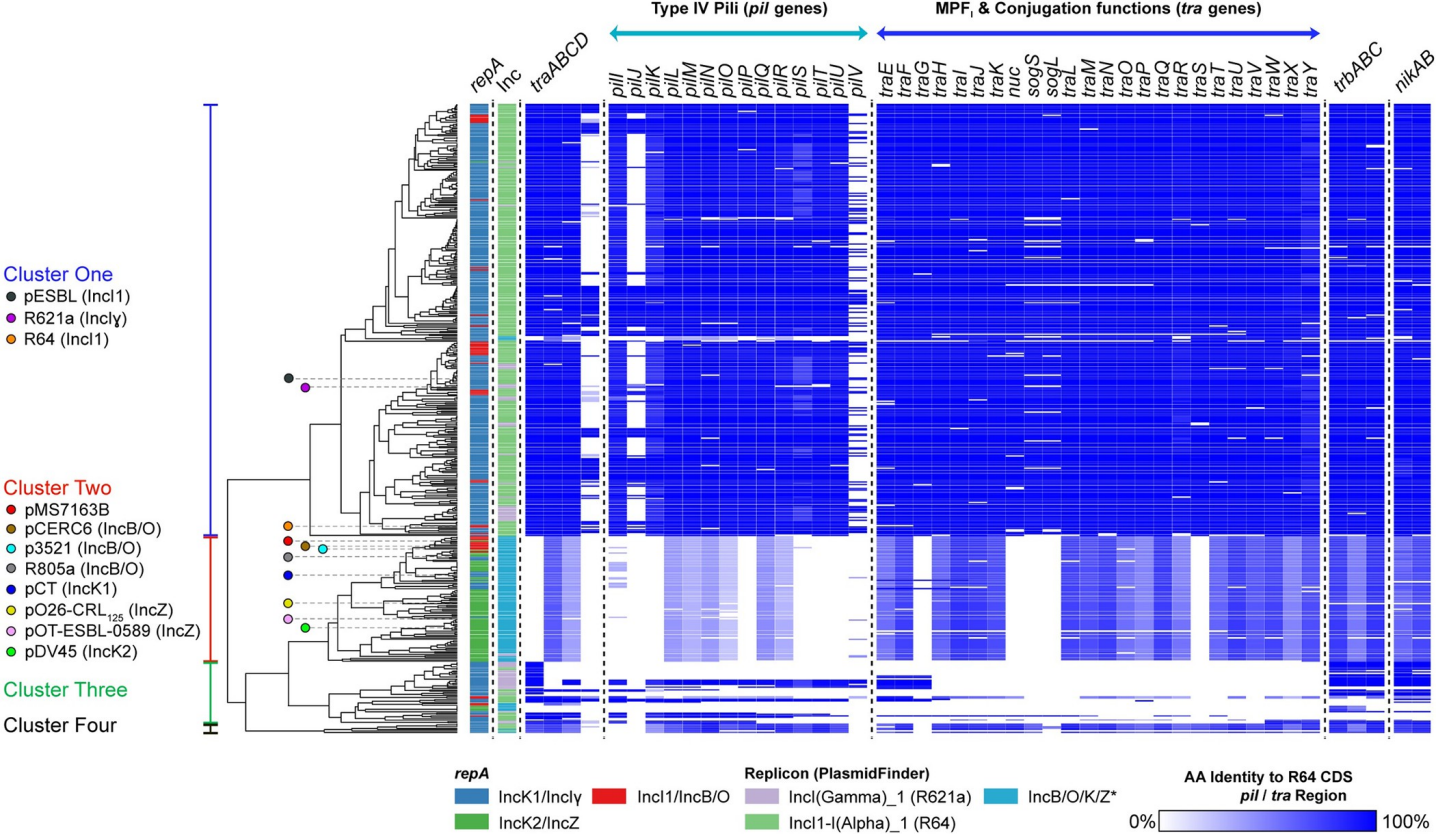
Cladogram of 460 I-complex Plasmids. To generate the cladogram, unique ORFs from all 460 plasmids were first combined into a hypothetical plasmid. Using sequence similarity searches against the hypothetical plasmid at an 80% nucleotide sequence identity and length threshold, a binary sequence denoting ORF presence/absence for each plasmid was generated. All binary sequences were subjected to hierarchical clustering using Manhattan distance and visualized as a midpoint-rooted cladogram. This method was adapted from an ORF-based binarized structure network analyses tool [31]. The cladogram was arranged into four clusters based on hierarchical clustering and the total within sum of square method. The cladogram was annotated with the following metadata of interest: *repA* variant (IncK2/IncZ, IncI1/IncB/O, or IncIɣ/IncK1), PlasmidFinder Inc group assignment, and amino acid identity (%) against 47 R64 conjugation-associated proteins based on tBLASTn. The following reference plasmids were annotated: pESBL (IncI1; NC_018659.1), R621a (IncIɣ; NC_015965), R64 (IncI1; NC_005014), pCERC6 (IncB/O; MH287044), p3521 (IncB/O; NC_014843), R805a (IncB/O; MK088173), pCT (IncK1; NC_014477), pO26-CRL_125_ (IncZ; NC_022996), pOT-ESBL-0589 (IncZ; MN335640), pDV45 (IncK2; KR905384). Plasmid pMS7163B belongs to cluster two (IncB/O/K/Z) closest to the IncB/O reference plasmids. The 460 I-complex plasmids were isolated from the following: *E*. *coli* (332/460); *Salmonella enterica* (90/460); *Shigella sonnei* (13/460); *Klebsiella pneumoniae* (9/460); *Shigella dysenteriae* (5/460); other *Salmonella* sp. (3/460); other *Escherichia* sp. (3/460); *Shigella flexneri* (1/460), uncultured bacterium (1/460).

**Fig 2:**
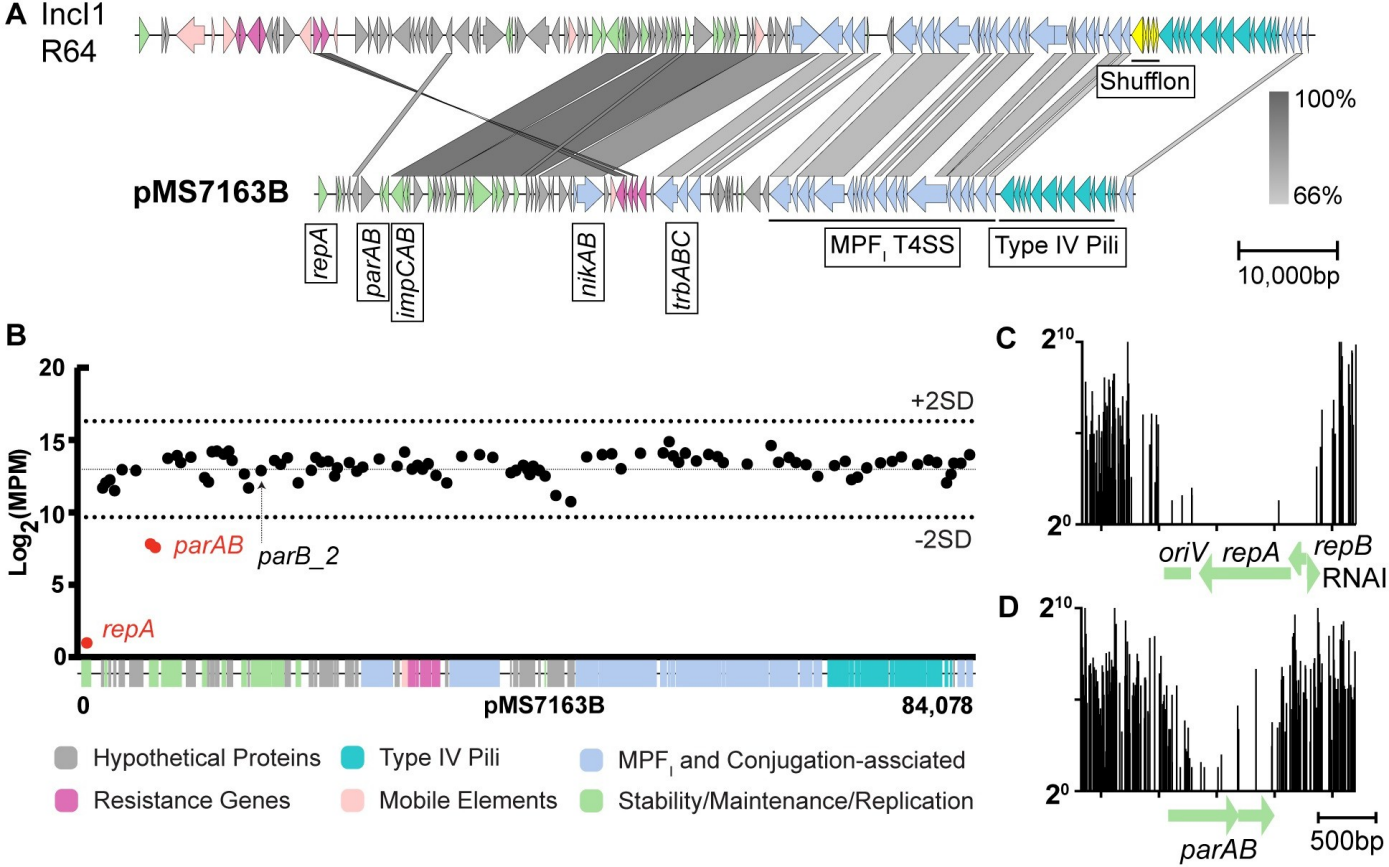
Sequence comparison of pMS7163B and genes required for replication/maintenance. **(A) Comparison of IncI1 reference plasmid R64 to pMS7163B.** Coding sequences (CDS) are shown, with arrowheads indicating gene orientation. Important features are labelled below the sequence. Colour gradient between plasmids is indicative of nucleotide sequence conservation (%) generated using BLASTn with a minimum sequence length of 500 bp. The figure was generated using EasyFig [32]. **(B) Number of insertions in each CDS across pMS7163B.** Insertion count is represented as Log_2_(Mutants per Million—MPM). Black dots represent CDS with Log_2_(MPM) values within the Mean ± 2SD thresholds and red dots represent CDS with Log_2_(MPM) values below the Mean– 2SD threshold, which are defined as required for plasmid replication/maintenance. **Enlarged view of the reads mapped to the (C) replicon and (D) *parAB* regions.** Log_2_ (Raw Read Counts) on the y-axes represent mini-Tn*5*-Cm insertions.

We established a dataset of 460 I-complex plasmids from public databases and examined their relatedness using an ORF-based binarized structure network analyses tool (Fig 1). Two major clusters were identified, one that contained IncI1/IncIɣ plasmids (cluster 1; 69% of plasmids) and a second that contained IncB/O/K/Z plasmids (cluster 2; 20% of plasmids). This grouping was largely congruent with clustering based on the *repA* gene and PlasmidFinder (replicon-RNAI) typing (Fig 1). Plasmid pMS7163B belongs to cluster 2, closest to the IncB/O reference plasmids, and contains a replicon identical to the IncZ plasmid pOT-ESBL-0589 [7]. There was very low sequence conservation of genes associated with conjugation between plasmids from each cluster. While plasmids of cluster 1 share ~100% conservation with most R64 conjugation genes, plasmids of cluster 2 have low-to-undetectable conservation across the *pil* region and weak (~50%) conservation of the *tra/trbABC/nikAB* genes. Indeed, a pairwise comparison between plasmid R64 (cluster 1; IncI1) and plasmid pMS7163B demonstrates that although the conjugation-associated regions of both plasmids are similarly organised, they share very low nucleotide sequence conservation (Fig 2A). Plasmids in cluster 3 exhibited the greatest divergence and had low-to-undetectable conservation of most predicted conjugation genes, suggesting either a loss of conjugation genes and/or a distantly evolved conjugation region. Plasmids in cluster 4 were rare in the dataset and their predicted conjugation regions were most similar to plasmids from cluster 1. Notably, *repA* and replicon types were unexpectedly shifted between different clusters of the cladogram, suggesting recombination between plasmids from different clusters. The remainer of this study focussed on analysis of pMS7163, a hybrid I-complex plasmid containing an IncB/O backbone with an IncZ replicon that is representative of plasmids in cluster 2 (Fig A in S1 Text), with the primary objective to define genes involved in replication/maintenance and conjugation.

### Identification of genes required for pMS7163B replication/maintenance

Plasmid pMS7163B is an 84,078 bp conjugative plasmid containing 97 predicted CDSs, including genes encoding a MPF_I_ T4SS (*tra* and *trb* operons), type IV pilus (*pil* genes), MOB_P_ relaxosome (*nikAB*), transfer regulators (*traBC*) and resistance to trimethoprim (*dfrA14*) and sulfonamides (*sul2*) (Fig B in S1 Text). To identify genes required for replication/maintenance, plasmid pMS7163B was initially subjected to *in vitro* miniTn*5*-Cm mutagenesis, generating a highly saturated transposon mutant library that was subsequently transformed into *E*. *coli* TOP10 to achieve ~18,000 mutants. DNA was extracted from the pooled mutants and subjected to TraDIS analyses, which identified 14,868 unique insertion sites, equivalent to an average of one insertion every 5.65bp across the plasmid. Next, the read counts for each gene were normalized to Log_2_(Mutants per Million—MPM), with the prediction that genes essential for these processes would be lost during replication and reflected as genes with low Log_2_MPM values. Two genetic units were identified, the replicon region and a *parAB* partitioning system (Fig 2). The *parAB*_pMS7163B_ genes share no detectable nucleotide conservation or amino acid identity to the *parAB* partitioning systems of R64 (IncI1) and R621a (IncIɣ), and low amino acid identity to the partitioning system of pND11_107 (IncI1). A query against the 460 I-complex plasmids database revealed that 48% of plasmids carried *parAB*_R64,_ 20% carried *parAB*_pMS7163B_, 7% carried both *parAB*_R621a_ and *parAB*_pMS7163B_, 6% carried *parAB*_pND11_107_, and only a single plasmid carried *parAB*_R621a_ alone (Fig C in S1 Text). The remaining ~15% plasmids did not carry any of the above partitioning systems. Plasmid pMS7163B contains an additional putative partitioning gene, referred to as *parB_2*. However, *parB_2* was not required for plasmid replication/maintenance under the conditions employed in our experiments (Fig 2B).

### Identification of genes involved in pMS7163B surface conjugation

**Fig 3:**
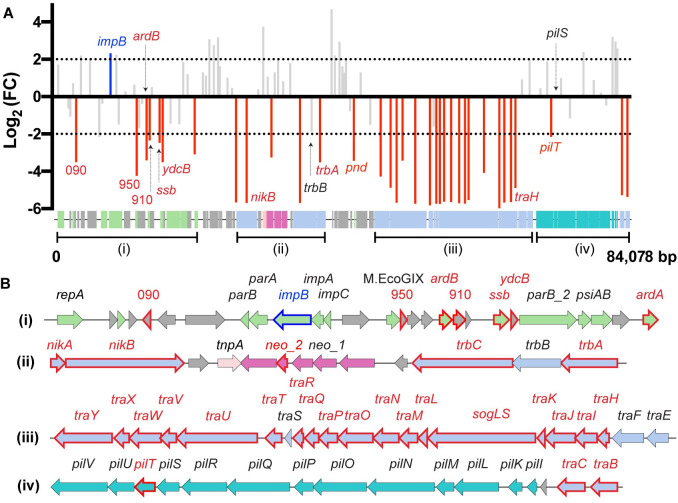
Plasmid conjugation genes. **(A) Plasmid pMS7163B conjugation genes as defined by TraDIS.** Log2(fold-change–FC) values for insertions in each gene between the pre- and post-conjugation libraries are displayed against pMS7163B. Genes required for conjugation (LogFC ≤ -2; false discovery rate–FDR ≤ 0.001) are red bars. Genes predicted to repress conjugation (LogFC ≥ 2; FDR ≤ 0.001; Read count at any site not exceeding 30% of total reads within the gene) are blue bars. Non-conjugation genes are grey bars. Genes selected for validation are labelled. **(B) Regions implicated in conjugation.** Genes required for conjugation are labelled in red font with a red-bordered arrow. Plasmid pMS7163B is colour coded based on predicted function: Green–stability/maintenance/replication; Blue–MPF_I_ and conjugation associated; Teal–Type IV pili biogenesis; Dark pink–Resistance; Light pink–Mobile elements; Grey–Hypothetical/Others. The *pnd* gene is located outside of regions I-IV.

To identify pMS7163B genes required for surface conjugation, we performed an experiment involving transfer of the mutant plasmid library (TOP10 + pMS7163B::mini-Tn*5*-Cm; pre-conjugation library) to the recipient *E*. *coli* strain J53 with 1:10 donor to recipient ratio at 37°C. These optimized conditions were validated experimentally, with donor:recipient ratio, temperature and donor strain impacting transfer frequency (Fig D in S1 Text). Two independently generated transconjugant pools (J53 + pMS7163B::mini-Tn*5*-Cm; post-conjugation library) were then subjected to TraDIS analysis. Total read counts in each CDS were compared between the pre-conjugation and post-conjugation libraries to obtain Log_2_(fold-change) (LogFC) values. Genes with a LogFC ≤ -2 were defined as required for conjugation (false discovery rate ≤ 0.001) while genes with a LogFC ≥ 2 and with a read count at any insertion site not exceeding 30% of the total reads within the gene were considered repressors of conjugation (the 30% threshold was set to exclude pre-existing insertion biases within the pre-conjugation pool; Fig E in S1 Text). A total of 35 genes were required for conjugation and one gene (*impB*) was considered to repress conjugation (Fig 3A). These genes are largely located in three distinct regions of pMS7163B, and were separated into the following categories based on predicted function: (i) regulators of transfer gene expression–*traBC*; (ii) type IV pilus biogenesis–*pilT*; (iii) MPF_I_ T4SS and conjugation functions–*traHIJKLMNOPQRTUVWXY*, *sogLS*, *trbAC*; (iv) MOB_P_ relaxosome–*nikAB*; and (v) genes not previously associated with conjugation–*impB*, 090, *pnd*, *neo_2*, *ardA*, *ydcB*, *ssb*, 910, *ardB*, 950 (Fig 3B). A complete list of identified CDSs and Log_2_FC values is presented in Table A of S1 Table.

### Validation of genes involved in pMS7163B surface conjugation

**Fig 4:**
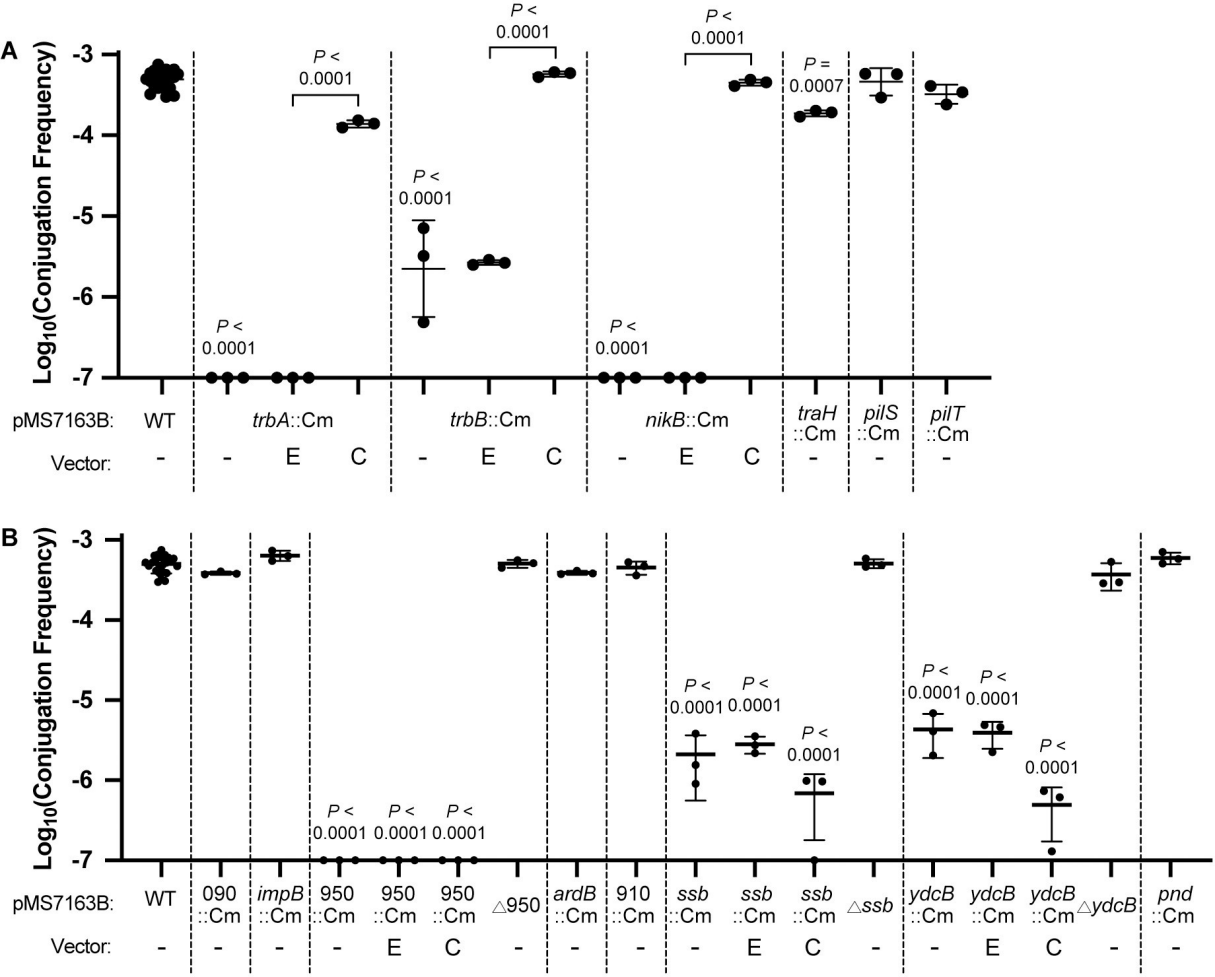
**Validation of pMS7163B conjugation genes (A) Conjugation frequencies of wildtype pMS7163B, *trbA*::Cm, *trbB*::Cm, *nikB*::Cm, *traH*::Cm, *pilS*::Cm, *pilT*::Cm and their complemented strains. (B) Conjugation frequencies of wildtype pMS7163B, 090::Cm, *impB*::Cm, 950::Cm, Δ950, *ardB*::Cm, 910::Cm, *ssb*::Cm, Δ*ssb*, *ydcB*::Cm, Δ*ydcB*, *pnd*::Cm and their complemented strains.** Vector complementation:–, no vector; E, empty pSU2718; C, pSU2718 with complemented gene. Conjugation frequency is represented as three biological replicates of Mean ± SD of transconjugants/donor. Data for wildtype pMS7163B comprises 23 biological replicates performed in triplicate. One-way ANOVA and Sidak’s multiple comparisons were performed on log_10_ transformed values.

To validate the role of genes identified by TraDIS, we constructed two sets of defined mutants and tested their capacity for surface conjugation compared to WT pMS7163B, using MG1655 as the donor strain (Fig 4). All mutants were constructed by replacing the gene of interest with a chloramphenicol (Cm) cassette in the native orientation using λ-Red recombineering.

The first set of mutants consisted of genes known to be associated with conjugation in R64 as follows: required for surface conjugation–*trbA*, *nikB*; reduced transfer activity when deleted–*trbB*; not required for surface conjugation–*traH*; required for liquid conjugation only–*pilS*, *pilT* (Fig 4A). The *trbA*::Cm and *nikB*::Cm mutants were unable to conjugate (Log_10_Conjugation Frequency < -7), and complementation *in trans* restored conjugation ability. For *trbB*, while the TraDIS data approached significance (Fig 3A; LogFC = -1.88), the defined *trbB*::Cm mutant plasmid exhibited significantly reduced conjugation (Fig 4A). The *traH* gene was identified in our study as required for conjugation (Fig 3A; LogFC = -4.90), but is not required for conjugation in R64 [20]. Upon validation, the *traH*::Cm mutant had a small but significant reduction in conjugation frequency (Fig 4A). The TraDIS classification of the remaining *tra* genes is congruent with R64 data [20]. The mutants *pilS*::Cm and *pilT*::Cm did not show a significant reduction in surface conjugation frequency (Fig 4A), indicating that surface conjugation does not require the type IV pili. The *pilT* gene is likely a false positive hit by TraDIS (borderline LogFC value of -2.16). Overall, our TraDIS data demonstrated a requirement for the MPF_I_ T4SS and relaxosome but not the type IV pili for pMS7163B surface conjugation.

The second set of mutants consisted of eight uncharacterised genes that were not previously associated with conjugation (*impB*, 090, *pnd*, *ydcB*, *ssb*, 910, *ardB*, 950). Conjugation experiments revealed three of the eight mutants exhibited an altered transfer frequency; mutant plasmids pMS7163B *ydcB*::Cm and *ssb*::Cm demonstrated a reduction in conjugation frequency while mutant plasmid pMS7163B 950::Cm was unable to conjugate (Fig 4B). Unexpectedly, complementation of the respective genes did not restore conjugation frequency. To eliminate any possible polar effects of the Cm cassette insertion, we removed the Cm cassette and repeated the conjugation assay. Removal of the Cm cassette for mutant plasmids pMS7163B *ydcB*::Cm, *ssb*::Cm, and 950::Cm restored their conjugation frequency to WT level, suggesting polar effects of the Cm cassette.

The five mutants with no change in conjugation frequency (090, *impB*, *ardB*, 910, *pnd*) were additionally validated using *E*. *coli* TOP10 as an alternative donor. In contrast to the results using MG1655 as a donor, we observed a significant decrease in conjugation frequency for *ardB*::Cm and 910::Cm (Fig F in S1 Text). The genes *ardB* and 910 thus exhibited donor-specific conjugative roles, affecting conjugation frequency from TOP10 but not MG1655.

### Identification of pMS7163B genes that adversely affect host fitness

**Fig 5:**
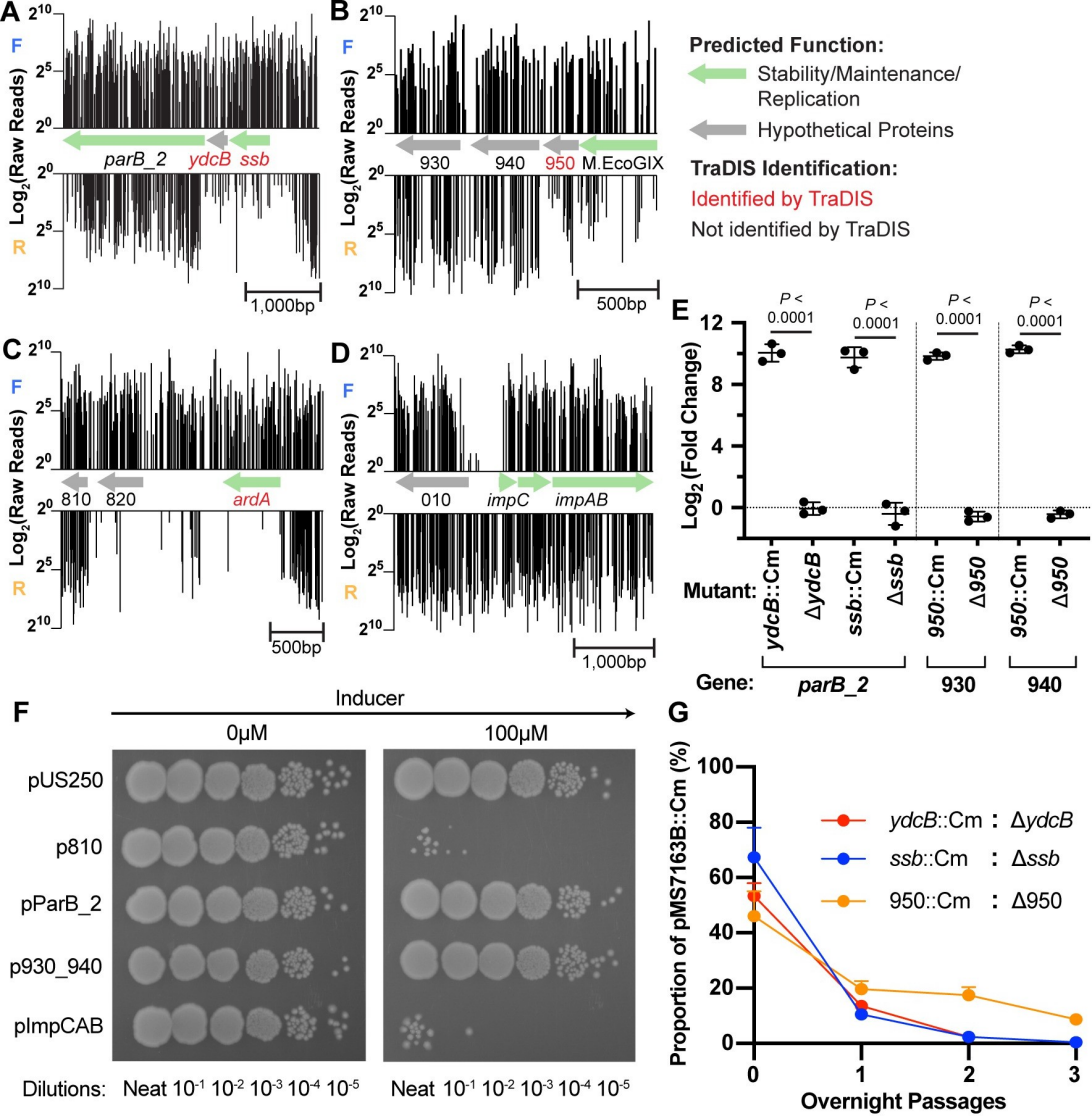
**Transposon reads mapped to: (A) *ydcB* and *ssb*; (B) 950; (C) *ardA* (D) *impCAB*.** Log_2_(Raw Reads) on the y-axes represent the number of reads mapped to each mini-Tn*5*-Cm insertion with the promoter orientated in the same direction as the forward strand (top graphs indicated as F), or with the promoter orientated in the direction of the reverse strand (bottom graphs indicated as R) from the first pre-conjugation library replicate. **(E) RT-qPCR analyses of *parB_2*, 930 and 940 expression normalised against the pMS7163B replication initiation gene *repA*.** Data is shown as Mean ± SD of three biological replicates. One-way ANOVA and Sidak’s multiple comparisons were performed on log_2_-transformed values. **(F) Serial dilutions of MG1655(pMS7163B) containing pUS250 (control) or p810, pParB_2, p930_940, or pImpCAB.** Overnight cultures were standardized to OD_600_ 2.0, serially diluted tenfold, and spotted onto LB agar + trimethoprim + kanamycin, with and without the presence of cumic acid inducer (100μM). Photos were taken after overnight incubation at 37°C and are representative of three biological replicates. **(G) Proportion of *ydcB*::Cm, *ssb*::Cm, and *950*::Cm mutants (%) in a mixed growth assay.** Each Cm-carrying mutant was mixed at a 50:50 ratio with its corresponding Cm-removed mutant and the percentage of the Cm-carrying mutant in the mix was measured at the end of each consecutive overnight passage for 3 days. Each passage was incubated for 14–16 hours at 37°C and 250 rpm shaking with LB + trimethoprim, then transferred to the next passage by diluting 1:100. Data is shown as Mean ± SD of three biological replicates.

It has been previously demonstrated that the Cm cassette in our mini-Tn*5* transposon can drive the transcription of a downstream gene if the insertion position is favourable [13, 29, 33, 34]. The same Cm cassette was also used to generate the targeted *ydcB*::Cm, *ssb*::Cm and 950::Cm mutations in pMS7163B. Therefore, we hypothesised that the reduction in conjugation frequency was not caused by insertional inactivation of these genes but instead was due to the overexpression of the respective downstream gene. Indeed, we identified an orientation bias in mini-Tn*5*-Cm insertion and read count in the pre-conjugation library in the region upstream of these coding sequences (Fig 5A and 5B). Insertions with the Cm promoter in the same orientation as the downstream gene were found with lower frequency compared to insertions with the Cm promoter in the opposite orientation. This pre-conjugation library insertion pattern suggested that overexpression of the downstream genes via Cm promoter readthrough affected either host fitness or plasmid stability/maintenance, and these effects were likely exacerbated during conjugation experiments (hence their identification as required for conjugation). Further examination of the pre-conjugation libraries identified two additional areas with a similar insertion bias, upstream of 810 (Fig 5C) and *impCAB* (Fig 5D), suggesting adverse effects upon their overexpression. Reverse transcription-quantitative PCR (RT-qPCR) confirmed increased transcription of the genes *parB_2* (downstream of *ydcB* and *ssb*) and 930/940 (downstream of 950) in the pMS7163B::Cm mutants compared to their respective Cm-removed mutants (Fig 5E).

To investigate the impact of the genes 810, *parB_2*, 930/940, and *impCAB* on the host cell, we cloned these genes into the tightly controlled inducible expression vector pUS250 and transformed the resultant plasmids into MG1655 and MG1655(pMS7163B). When grown on LB agar with induction, the expression of 810 and *impCAB* resulted in a severe growth defect, while the expression of *parB_2* and 930/940 did not result in a noticeable phenotype (Fig 5F). These results were identical in the MG1655-only background (Fig G in S1 Text). Because expression of *parB_2* and 930/940 via pUS250 did not show any altered growth phenotype, we tested the impact of their overexpression on pMS7163B using mixed-growth competitive assays. The strains MG1655 + pMS7163B *ydcB*::Cm and pMS7163B *ssb*::Cm (both overexpressing *parB_2*), as well as pMS7163B 950::Cm (overexpressing 930/940), were mixed with their respective Cm-removed mutants at a 50:50 ratio and measured over three overnight passages. All three mutants carrying the Cm cassette were rapidly outcompeted after a single overnight passage (Fig 5G), suggesting that the effects of *parB_2* and 930/940 could be pMS7163B-dependant. Thus, the genes 810, *parB_2*, 930/940, *impCAB* impact host fitness by causing either severe growth defects or rapid out-competition within a mixed population when overexpressed, indicating their expression is controlled in pMS7163B.

### The 810, *parB_2*, 930/940 and *impCAB* genes are broadly conserved

**Fig 6:**
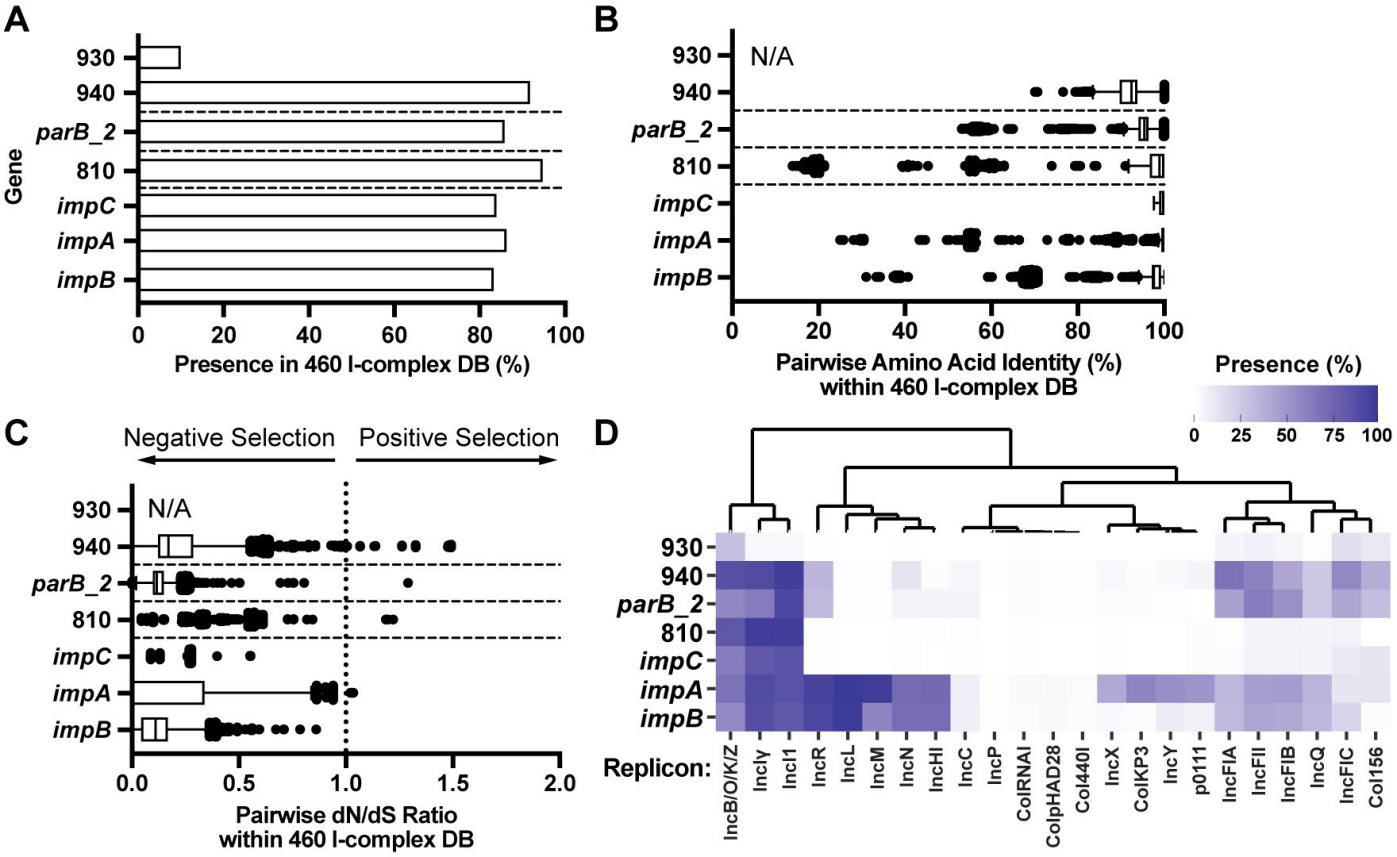
Conservation and sequence analyses of the genes *impCAB*, 810, *parB_2*, 930, and 940. **(A) Presence (%) in the 460 I-complex plasmid database. (B) Amino acid identity (%) between homologs. (C) Ratio of nonsynonymous (dN) to synonymous (dS) substitutions between homologs.** Coding sequences from pMS7163B were used as a tBLASTn query against 460 I-complex plasmids using an 80% query length threshold to identify homologs. Amino acid identity comparisons were performed using Clustal Omega and dN/dS ratios were estimated using pal2nal and PAML v4.9. Comparisons with dS < 0.01 or > 2 were excluded from analyses due to unreliable dN/dS estimations. Data for **(B)** and **(C)** are represented using Tukey’s boxplot, where the box limits represent first and third quartiles, the internal line represents median, and whiskers represent data within a ±1.5 interquartile range. Dots represent data outside of the whisker range. **(D) Heatmap of presence (%) within PLSDB plasmid database.** Replicons associated with <50 plasmids in the database and <5% presence for all genes of interest were removed from the heatmap.

To investigate the conservation of these genes with adverse impacts upon overexpression within the I-complex, the coding sequences from pMS7163B were used in a tBLASTn query against the I-complex plasmid database to identify homologs. Subsequently, we analysed the amino acid sequence divergence of these homologs by calculating amino acid percent identity and the ratio of nonsynonymous (dN) to synonymous (dS) substitutions between all possible pairs. All genes (with the exception of 930) were highly conserved within the I-complex, with more than 80% of all I-complex plasmids carrying identifiable homologs (Fig 6A). As 930 was restricted to ~10% of the I-complex, we excluded it from further analyses. For the remaining genes, pairwise comparisons of the homologs revealed extremely high amino acid identity (Fig 6B; median > 90%) and low dN/dS ratios (Fig 6C; median_*impC*/*impA*/810_ = 0.001; median_*impB*_ = 0.1105; median_*parB_2*_ = 0.1163; median_940_ = 0.1708) indicating negative selection pressure. The amino acid identity and dN/dS data for all other broadly conserved pMS7163B coding sequences were also calculated, revealing a similar pattern of negative selection (Fig H in S1 Text).

We expanded our analyses outside the I-complex by querying these genes against the publicly available plasmid database PLSDB [35]. Homologs of the genes 940, *parB_2*, and *impAB* were prevalent in multiple incompatibility groups, with greatest association in IncR, IncF, IncQ, and Col156 plasmids (Fig 6D). The remaining genes 930, 810, and *impC* were mostly restricted to the I-complex, with 930 being identified in IncB/O/K/Z plasmids only. Overall, the conservation across a broad range of incompatibility groups is strong evidence that these plasmid-encoded genes play roles beneficial to the host and/or plasmid.

## Discussion

I-complex plasmids are an important conduit for the spread of antibiotic resistance in pathogenic *Enterobacteriaceae* [10, 11, 13, 15–17]. Despite this, there are limitations in the capacity of *in silico* typing methods to accurately capture and resolve the genetic diversity of I-complex plasmids. Currently, I-complex plasmids are typed using the PlasmidFinder tool [9], which assigns plasmids to an incompatibility group based on sequence similarity of a region upstream of *repA* (encompassing approximately half of the RNAI); these include IncI1 (prototype plasmid R64), IncIɣ (R621a), IncB/O/K/Z (pECOED; pO26-CRL; p3521; pCT). There are limitations with this method, as assignment can be based on nucleotide identity as low as ~80%, and low-identity amplicons likely have mutations in the RNAI region that could affect phenotypic incompatibility. Other *in silico* methods of I-complex classification are either limited to a sub-type (i.e. IncI1 pMLST scheme [36]) or have limited resolution (i.e. *repA* phylogeny [8]). Here, we utilized an ORF-based clustering approach to examine I-complex plasmid relatedness, which offers several advantages: (i) it is replicon-independent; (ii) it provides greater resolution compared to *repA*-based phylogeny alone; (iii) it has no requirement for specific loci; and (iv) it can be applied to all I-complex plasmids. Our approach clearly separated IncI1/IncIɣ and IncB/O/K/Z plasmids based on plasmid content. Further resolution within these sub-clusters is difficult to achieve and would require an understanding of all members of the I-complex, which prior to this study was narrowly focussed on IncI1 plasmids, particularly R64.

The IncI1 R64 plasmid is generally used to predict the function of proteins on distantly related I-complex plasmids via sequence homology [2, 16, 19–23, 37]. However, limited experimental evidence supports these inferences, which become increasingly imprecise with lower identity. Here, we utilized TraDIS to simultaneously identify all genes required for the replication/stability and surface conjugation of the hybrid I-complex plasmid pMS7163B, which is distantly related to R64 as demonstrated by our ORF-based clustering analysis. Plasmid pMS7163B contains two putative partitioning systems, and we provide experimental evidence to demonstrate that active partitioning is mediated by *parAB*_pMS7163B_ rather than *parB_2*. Notably, the *parAB*_pMS7163B_ system is absent in R64, which contains an unrelated *parAB*_R64_ partitioning system that does not share any sequence conservation with *parAB*_pMS7163B_ but has been identified (with 100% sequence conservation) in the IncI1 plasmid pESBL [38, 39]. Other partitioning systems described in the I-complex that share little to no detectable sequence conservation with *parAB*_pMS7163B_ include that of the IncI1 plasmid pND11_107 [40, 41] and IncIɣ plasmid R621a [17]. Notably, only ~85% of the 460 I-complex plasmids have at least one of the aforementioned partitioning systems (Fig C in S1 Text), suggesting additional partitioning systems important to I-complex plasmids remain to be identified. The use of high-resolution transposon-based methodologies such as TraDIS and Tn-seq, as demonstrated here and in previous studies to genetically characterise IncI1 [38], IncC [42, 43], and IncF [44] plasmids, provide a tractable methodology to decipher the function of genes with predicted functional redundancy, and identify new genes involved in I-complex plasmid replication and stability.

Conjugation is one of the most important mechanisms used by bacteria to transfer antibiotic resistance genes. Here, we identified roles for the MPF_I_ T4SS and the relaxosome in pMS7163B surface conjugation. One difference in the conjugation requirements between pMS7163B and R64 is *traH*; this gene was required in pMS7163B (this study) but is not required in R64 [20]. Mutation of *traH* in pMS7163B led to a small but significant reduction in conjugation, and consistent with our data *traH* was also shown to be required for surface conjugation in the IncI1 plasmid pESBL [38]. The genes encoding type IV pili, which are predicted to mediate cell-cell contact during liquid mating, did not contribute to surface conjugation. Unfortunately, we were unable to apply our methodology to examine liquid conjugation as the transfer frequency of plasmid pMS7163B was too low under this condition (~2.17 x 10^−6^ transconjugants/donor) to obtain a representative post-conjugation library. Notably, under the conditions of our study, no repressors of conjugation were identified. While *impB* was implicated as a potential conjugation repressor (Fig 3A), mutation of *impB* did not increase conjugation frequency (Fig 4B).

Our study employed the cloning strain TOP10 to generate the pre-conjugation library due to its high transformation efficiency, and we used MG1655 as the donor in subsequent validation experiments. This strategy enabled the identification of two genes (*ardB* and 910) that played donor-specific roles, affecting conjugation from TOP10 as donor but not MG1655. ArdB has anti-restriction activity, which protects incoming plasmid DNA from restriction by the chromosomally-encoded EcoKI type I restriction-modification system [45]. EcoKI methylates recognition sites on host DNA, but cleaves unmethylated DNA from mobile genetic elements such as phage and conjugative plasmids [46]. EcoKI is absent in TOP10 (Δ*hsdRMS*) but present in MG1655 and its derivative J53. Accordingly, conjugation from TOP10 to J53 in the absence of ArdB likely results in EcoKI_J53_-mediated restriction of pMS7163B *ardB*::Cm; noting that pMS7163B contains 15 predicted EcoKI restriction sites (Fig B in S1 Text), thus decreasing successful conjugative events. As MG1655 and J53 both have functional EcoKI, the restriction barrier is not present [47–49]. The function of the coding sequence 910, which does not share significant sequence conservation with any other functionally characterised gene in the NCBI database, remains to be elucidated.

The TraDIS analysis identified several uncharacterised genes co-located in the leading transfer region of pMS7163B (*impCAB*, 810, *parB_2*, 930/940), all of which exhibited a miniTn*5*-Cm insertion orientation bias in the region upstream of their coding sequence. We hypothesized that this bias was caused by the introduction of a strong promoter driving expression of these genes and confirmed this by demonstrating an impact on host cell fitness. In the case of the coding sequence 810 and the *impCAB* genes, overexpression resulted in a severe growth defect independent of the presence of pMS7163B (Figs 5 and G in S1 Text), suggesting a link to host cell toxicity and/or plasmid stability. Previous work has shown the *impCAB* genes contribute to DNA repair following UV damage [50–52], while the 810 coding sequence does not exhibit significant similarity to any functionally characterized protein. In contrast, overexpression of the *parB_2* and 930/940 genes only resulted in a phenotype when driven by an upstream Cm cassette insertion on pMS7163B, resulting in rapid out competition in a mixed competitive assay. The *parB_2* gene encodes a putative Type I partitioning protein, and we hypothesize that *cis* overexpression of *parB_2* interferes with the native *parAB*_pMS7163B_ partitioning system, resulting in pMS7163B destabilisation and decreased fitness within a population. The broad conservation of *parB_2* across multiple Inc groups (Fig 6D) suggests a poorly understood but important role in plasmid segregation or partitioning that remains to be explored. The proteins encoded by 930 and 940 do not share significant identity with any functionally characterized proteins and their functions remain to be elucidated. Overall, our discovery that overexpression of these broadly conserved genes negatively influences plasmid stability/maintenance or early conjugation, as well as host fitness, identifies their important role in the biology of antibiotic resistance-associated I-complex plasmids.

## Materials and methods

### Bacterial strains and growth conditions

A full list of strains used in this study are listed in Table B of S1 Table. All strains were routinely cultured at 37°C in either liquid or solid lysogeny broth (LB) under shaking (250 rpm) or static conditions, supplemented with appropriate antibiotics at the following concentrations: chloramphenicol (30μg/mL), kanamycin (50μg/mL), trimethoprim (100μg/mL), sodium azide (100μg/mL), gentamicin (20μg/mL) unless otherwise stated. Induction of pUS250-based constructs was performed by the addition of cumic acid to a final concentration of 100μM (Sigma-Aldrich; 268402-5G). All strains were stocked at -80°C in 15% glycerol. Plasmid pUS250 was a gift from Nicholas Coleman (Addgene plasmid # 198322; http://n2t.net/addgene:198322; RRID: Addgene_198322).

### DNA purification and analyses

Plasmid pMS7163B was extracted from MS7163 using the PureLink HiPure Midiprep Plasmid DNA Purification Kit (Invitrogen). All other plasmids were extracted using the QIAprep Spin Miniprep Kit (Qiagen). Genomic DNA was extracted using the Ultraclean Microbial DNA Isolation Kit (Qiagen). DNA was quantified using either a NanoDrop 2000 (Thermo Scientific) or Qubit 2.0 Fluorometer (Life Technologies).

### PCR and sequencing

DNA fragments for cloning and mutations were amplified using KAPA HiFi polymerase (Roche). Colony PCRs were performed using OneTaq DNA polymerase (New England Biolabs). Sanger sequencing reactions were prepared using BigDye Terminator Mix v3.1 and sequenced by the Genetic Research Services, UQ. A full list of primers used in this study can be found in Table C of S1 Table.

### Transformations and mutant generation

Electrocompetent cells were prepared, and transformations were performed as previously described [26]. After electroporation, cells were recovered in LB + 5mM MgCl_2_ [53]. All mutants were constructed using λ-Red-mediated homologous recombination as previously described [54]. All constructs were confirmed by Sanger sequencing.

### Surface conjugation assay

Overnight cultures of donor and J53 recipient were standardized (OD_600_ = 2.0) in fresh LB and mixed at various donor to recipient ratios (1:1, 1:2, 1:5, 1:10). Mating mixes were serially diluted tenfold in 0.9% NaCl, and 5μL of each dilution was spotted onto LB agar supplemented with appropriate antibiotics to select for donors, recipients, and transconjugants. Surface conjugation was allowed to proceed over 16 hours of incubation at 37°C on the agar surface, after which transconjugant colonies were enumerated. The conjugation frequency was expressed as the number of transconjugants/donor. Conjugation frequency values of pMS7163B mutants are available in Table G of S1 Table.

### RT-qPCR

Mating mixes (1:1 donor to recipient ratio) under surface conjugation conditions (37°C, 45 minutes) were stabilized in two volumes of RNAprotect Bacteria Reagent (Qiagen). Subsequent total RNA extraction, first-strand cDNA synthesis, RT-qPCR and data analyses were performed as previously described [55]. Transcript levels were normalised against the pMS7163B replication initiation gene *repA*, which displayed consistent cycle threshold values across all biological replicates (Fig I in S1 Text). One-way ANOVA and Sidak’s multiple comparisons were performed on log_2_-transformed values. Cycle threshold values for all genes are available in Table H of S1 Table.

### Mixed-growth competitive assay

Overnight cultures (LB + trimethoprim) of MG1655 + pMS7163B *gene*::Cm and Δ*gene* mutants were mixed at a 50:50 ratio at an OD_600_ of 0.1 in LB + trimethoprim. Mixed cultures were allowed to grow overnight (14–16 hours) under shaking conditions at 37°C in three consecutive passages. Each passage was transferred to the next using a 1:100 dilution. After each passage, cultures were serially diluted and spotted onto LB agar supplemented with the appropriate antibiotics to select for MG1655 + pMS7163B *gene*::Cm and total counts. Data is graphed as proportion of MG1655 + pMS7163B *gene*::Cm mutants in the culture, with the raw data being available in Table I of S1 Table.

### Plasmid pMS7163B *in vitro* transposon mutagenesis

Custom mini-Tn*5*-Cm transposons were constructed as previously described [27]. Plasmid pMS7163B (200ng) was incubated with an equimolar ratio of mini-Tn*5*-Cm transposons with 1U of EZ-Tn*5* Transposase (Epicentre) according to manufacturer’s instructions. 9μL of this reaction was mixed with 540μL of electrocompetent TOP10 cells and split to 9 equal volumes for electroporation. After recovery, cells were plated onto LB agar + chloramphenicol to select for pMS7163B::mini-Tn*5*-Cm mutants. Mutant colonies were subsequently pooled by scraping into LB, mixed with glycerol to a final concentration of 15%, and stored at -80°C.

### Preparation of J53 + pMS7163B::mini-Tn*5*-Cm post-conjugation library

The TOP10 + pMS7163B::mini-Tn*5*-Cm pre-conjugation library was mixed with overnight J53 recipient cells (standardized to OD_600_ 2.0) at a 1:10 donor to recipient ratio and spotted onto 0.22μm triton-free nitrocellulose membrane filter papers (Merck; GSTF01300) on LB agar and incubated at 37°C for 2 hours. Cells were dislodged in 0.9% NaCl by vortexing and plated onto LB agar + sodium azide + chloramphenicol to select for transconjugants. Transconjugant colonies were subsequently pooled by scraping into LB, mixed with glycerol to final concentration of 15%, and stored at -80°C. This was performed in duplicate to obtain two post-conjugation libraries.

### Library preparation and transposon directed insertion-site sequencing (TraDIS)

Library preparation was performed using the Illumina NexteraFlex DNA Prep Kit (Illumina) with modifications for TraDIS. Briefly, approximately 300ng of genomic DNA was fragmented and tagged with an adaptor sequence in a single step. An enrichment PCR targeting DNA fragments with a mini-Tn*5*-Cm cassette was performed using the custom Tn*5*-specific enrichment primer 4844 and supplied index 1 primers. The enrichment PCR was run on the following thermocycler program: 68°C for 3 minutes; 98°C for 3 minutes; 22 cycles of 98°C for 45 seconds, 62°C for 30 seconds, 68°C for 2 minutes; and a final 68°C for 1 minute. Libraries were subsequently cleaned according to manufacturer’s instructions. TraDIS was performed as previously described [42].

### TraDIS data analyses

Data analysis and insertion site mapping was performed as previously described [42]. Genes with a Log_2_(Mutants per Million) (Log_2_MPM) value two SDs below the mean were defined as required for plasmid maintenance/replication. MPM values are representative of insertion counts in a gene and was adapted from the RNA-Seq equivalent transcripts per million formula [56]. To identify conjugation-associated genes, gene insertion counts were compared between the pre- and post-conjugation libraries as previously described [43]. Genes with a Log_2_(Fold-change) (Log_2_FC) value of ≤ 2 with a false discovery rate (FDR) of ≤ 0.001 were defined as essential conjugation genes. The genes *neo_2* (truncated neomycin resistance gene assumed to be a false positive) and *ardA* (unable to obtain isogenic mutant) were excluded from further analyses. Genes with a Log2FC ≥ 2, an FDR of ≤ 0.001, and a read count at any site not exceeding 30% of the total reads within the gene were defined as genes repressing conjugation. The threshold of a read count at any site within a gene not exceeding 30% of the total reads mapped to that gene was implemented as previously described [30]. This was done to prevent pre-existing insertion biases within the pre-conjugation library from confounding the identification of true conjugational repressors.

### Construction of an I-complex plasmid database

A covariance matrix constructed from conserved sequences at the RNAI region [57] was used to query the PLSDB database (20,688 plasmids; 04/03/2020) [35], NCBI RefSeq database (12,638 plasmids; 02/08/2019) [58], and a collection of I-complex plasmids [8] using the cmsearch package included in INFERNAL (1.1.4) [59]. Plasmids with an e-value below the stringent threshold of 1x10e^-26^ were considered I-complex plasmids and were subsequently confirmed to contain an I-complex *repA* variant. No IncI2 plasmids were detected due to the lack of an RNAI homolog. Any plasmid matching a non-I-complex query against PlasmidFinder [9], less than 20kb, or containing multiple I-complex replicons were removed from further analyses. A total of 460 I-complex plasmids were isolated and evenly annotated with Prokka [60]. Details of the 460 I-complex plasmids, tBLASTn comparisons of conjugation-associated genes to R64 and pMS7163B are available in Tables D, E, and F of S1 Table, respectively.

### Bioinformatic analyses

Plasmid sequences were coloured using Artemis (18.0.3) [61], compared using BLASTn with a sequence length threshold of 500 bp and visualized using EasyFig [32]. A plasmid-backbone based cladogram of I-complex plasmids was constructed by collecting all unique ORFs into a single hypothetical plasmid. Each plasmid was subsequently used in a sequence similarity search against the hypothetical plasmid to generate a binary ORF presence/absence sequence for every plasmid as described in Suzuki, Doi (32). Binary sequences were subjected to hierarchical clustering based on Manhattan distance using the stats package in the R environment (4.0.4) [62], and visualized as a midpoint-rooted cladogram using the interactive Tree of Life [63]. Clustering was determined using the *fviz_nbclust* function (factoextra package 1.0.7; https://rpkgs.datanovia.com/factoextra/index.html) and the total within sum of square method. All plasmids were subsequently sorted into their respective clusters with the *cutree* function in the dendextend package (1.16.0) [64]. Analyses of gene presence/absence and homolog identification were performed using the appropriate BLAST executable [65]. PlasmidFinder amplicons were obtained from the PlasmidFinder database [9]. Alignments and amino acid identity comparisons were performed using Clustal Omega [66]. Codon alignments were generated using pal2nal v14 [67]. The ratio of nonsynonymous (dN) to synonymous (dS) substitutions were estimated using PAML v4.9 [68] with the following parameters: runmode = -2 (pairwise), model = 1, NSsites = 0. Pairs with dS < 0.01 or > 2.00 were removed from analyses due to unreliable dN/dS estimations [69].

## Supporting information

S1 Text **Fig A. Cladogram of 460 I-complex Plasmids.** This figure is similar to Fig 1 except for the amino acid identity (%) were compared against pMS7163B conjugation-associated sequences instead of those from R64. **Fig B. Genetic map of pMS7163B.** The rings represent the following from the outermost to the innermost: CDS on the forward strand; CDS on the reverse strand; GC-plot; GC-skew. Arrowheads indicate gene orientation. Plasmid pMS7163B is colour coded based on predicted function: Green–stability/maintenance/replication; Blue–MPF_I_ and conjugation associated; Teal–Type IV pili biogenesis; Dark pink–Resistance; Light pink–Mobile elements; Grey–Hypothetical/Others. Predicted EcoKI restriction sites (AACN_6_GTGC) are shown in orange. The figure was generated using Artemis (18.0.3). **Fig C. Distribution of *parAB* variants across the 460 I-complex plasmids.** The circular midpoint-rooted cladogram was based on ORF presence/absence using an ORF-based binarized structure network analyses tool and cut into four clusters based on hierarchical clustering and the total within sum of square method. Carriage of *parAB* variants was determined using a BLASTn search at an 80% query length threshold, with the following reference sequences used: pMS7163B (IncB/O backbone with IncZ replicon; CP026855), R64 (IncI1; NC_005014), R621a (IncIɣ; NC_015965), pND11_107 (IncI1; NC_019043). **Fig D. Characterization of pMS7163B surface conjugation frequency. (A) Effect of donor to recipient ratio on pMS7163B conjugation.** Plasmid pMS7163B was conjugated from *E*. *coli* MG1655 (donor) to *E*. *coli* J53 (recipient) at 37°C for 16 hours. **(B) Effect of temperature on pMS7173B conjugation.** Plasmid pMS7163B was conjugated from *E*. *coli* MG1655 (donor) to *E*. *coli* J53 (recipient) at a 1:1 donor to recipient ratio for 16 hours at 28°C, 37°C, and 43°C. **(C) Effect of host strain on pMS7163B conjugation.** Plasmid pMS7163B was conjugated from various *E*. *coli* donor strains to *E*. *coli* J53 (recipient) at multiple donor to recipient ratios for 16 hours at 37°C. Conjugation frequency for all experiments were calculated as transconjugants/donor. Data represents mean ± SD of three biological replicates. **Fig E. Transposon reads mapped to (A) 610 and (B) *tnpA* in both the (i) pre-conjugation and (ii) post-conjugation libraries.** Log_2_(Raw Reads) on the y-axes represent the number of reads mapped to each mini-Tn*5*-Cm insertion with the promoter orientated in the same direction as the forward strand (top graphs indicated as F), or with the promoter orientated in the direction of the reverse strand (bottom graphs indicated as R) from the first library replicate. Insertion sites that represent >30% reads mapped to their respective coding sequences in the post-conjugation library are shown in red with their proportions in %. The corresponding insertions in the pre-conjugation library are shown in green. **Fig F. Conjugation frequencies of TOP10 + wildtype pMS7163B, 090::Cm, *impB*::Cm, *ardB*::Cm 910::Cm, *pnd*::Cm**. Conjugation frequency is represented as three biological replicates of Mean ± SD of transconjugants/donor. One-way ANOVA and Sidak’s multiple comparisons were performed on log_10_ transformed values. **Fig G. (A) Growth curves of MG1655 + pMS7163B and (B) MG1655 carrying the following inducible expression vectors: (i) pUS250 (empty); (ii) p810; (iii) pParB_2; (iv) p930_940; (v) pImpCAB.** Overnight cultures were standardized to OD_600_0.05 and grown in LB + trimethoprim + kanamycin as appropriate. Induction was performed by the addition of cumic acid (100μM). Data represents mean ± SD of three biological replicates. **(C) Serial dilutions of MG1655 carrying the following inducible expression vectors: (i) pUS250 (empty); (ii) p810; (iii) pParB_2; (iv) p930_940; (v) pImpCAB.** Overnight cultures were standardized to OD_600_ 2.0, serially diluted tenfold, and spotted onto LB agar + kanamycin, with and without the presence of cumic acid inducer (100μM). Photos were taken after overnight incubation at 37°C and are representative of three biological replicates. **Fig H. Conservation and sequence analyses of broadly conserved genes. (A) Amino acid identity (%) between homologs in the 460 I-complex plasmid database. (B) Ratio of nonsynonymous (dN) to synonymous (dS) substitutions between homologs in the 460 I-complex plasmid database**. Coding sequences from pMS7163B were used as a tBLASTn query against 460 I-complex plasmids using an 80% query length threshold to identify broadly conserved genes. Amino acid identity comparisons were performed using Clustal Omega and dN/dS ratios were estimated using pal2nal and PAML v4.9. Comparisons with dS < 0.01 or > 2 were excluded from analyses due to unreliable dN/dS estimations. Data is represented using Tukey’s boxplot, where the box limits represent first and third quartiles, the internal line represents median, and whiskers represent data within a ±1.5 interquartile range. Dots represent data outside of the whisker range. Genes shown in red are genes that result in decreased host fitness when overexpressed on pMS7163B. **Fig I. Cycle threshold (CT) values for *repA* gene expression.** Data is representative of 135 technical replicates over 27 biological replicates, and is shown as Mean ± SD.
(DOCX)

S1 Table **Table A. LogFC of pMS7163B genes. Table B. Strains used in this study. Table C. Primers used in this study. Table D. I-complex plasmids used to construct database. Table E. Highest amino acid identities between conjugation-associated genes of the 460 I-complex plasmids and R64. Table F. Highest amino acid identities between conjugation-associated genes of the 460 I-complex plasmids and pMS7163B. Table G. Conjugation frequency of pMS7163B mutants. Table H. Cycle threshold values for RT-qPCR of *repA*, *parB_2*, 930, and 940**. **Table I. Proportion of pMS7163B::Cm mutants in a competitive growth assay.**
(XLSX)
