# Supplementary material for: Genetic basis of I-complex plasmid stability and conjugation
Source: PLoS Genet. 2023 Jun 22;19(6):e1010773. doi: 10.1371/journal.pgen.1010773 (PMC10286972; doi:10.1371/journal.pgen.1010773)
Supplement: S1 Text — Fig A. Cladogram of 460 I-complex Plasmids. This figure is similar to Fig 1 except for the amino acid identity (%) were compared against pMS7163B conjugation-associated sequences instead of those from R64. Fig B. Genetic map of pMS7163B. The rings represent the following from the outermost to the innermost: CDS on the forward strand; CDS on the reverse strand; GC-plot; GC-skew. Arrowheads indicate gene orientation. Plasmid pMS7163B is colour coded based on predicted function: Green–stability/maintenance/replication; Blue–MPFI and conjugation associated; Teal–Type IV pili biogenesis; Dark pink–Resistance; Light pink–Mobile elements; Grey–Hypothetical/Others. Predicted EcoKI restriction sites (AACN6GTGC) are shown in orange. The figure was generated using Artemis (18.0.3). Fig C. Distribution of parAB variants across the 460 I-complex plasmids. The circular midpoint-rooted cladogram was based on ORF presence/absence using an ORF-based binarized structure network analyses tool and cut into four clusters based on hierarchical clustering and the total within sum of square method. Carriage of parAB variants was determined using a BLASTn search at an 80% query length threshold, with the following reference sequences used: pMS7163B (IncB/O backbone with IncZ replicon; CP026855), R64 (IncI1; NC_005014), R621a (IncIɣ; NC_015965), pND11_107 (IncI1; NC_019043). Fig D. Characterization of pMS7163B surface conjugation frequency. (A) Effect of donor to recipient ratio on pMS7163B conjugation. Plasmid pMS7163B was conjugated from E. coli MG1655 (donor) to E. coli J53 (recipient) at 37°C for 16 hours. (B) Effect of temperature on pMS7173B conjugation. Plasmid pMS7163B was conjugated from E. coli MG1655 (donor) to E. coli J53 (recipient) at a 1:1 donor to recipient ratio for 16 hours at 28°C, 37°C, and 43°C. (C) Effect of host strain on pMS7163B conjugation. Plasmid pMS7163B was conjugated from various E. coli donor strains to E. coli J53 (recipient) at multiple donor to recipient ra [file pgen.1010773.s001.docx]

**
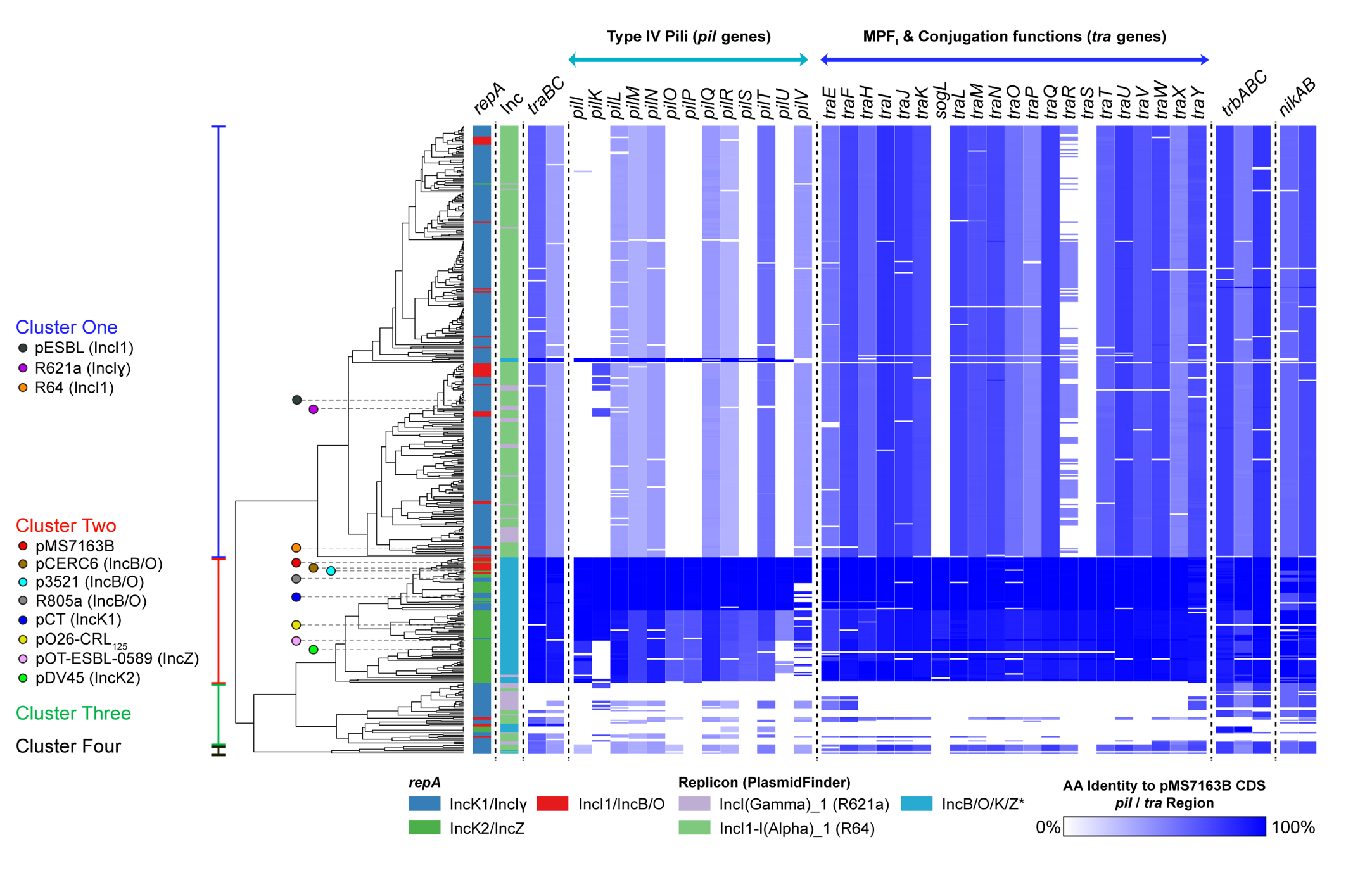
**

**Fig A. Cladogram of 460 I-complex Plasmids.** This figure is similar to Fig 1 except for the amino acid identity (%) were compared against pMS7163B conjugation-associated sequences instead of those from R64.

**
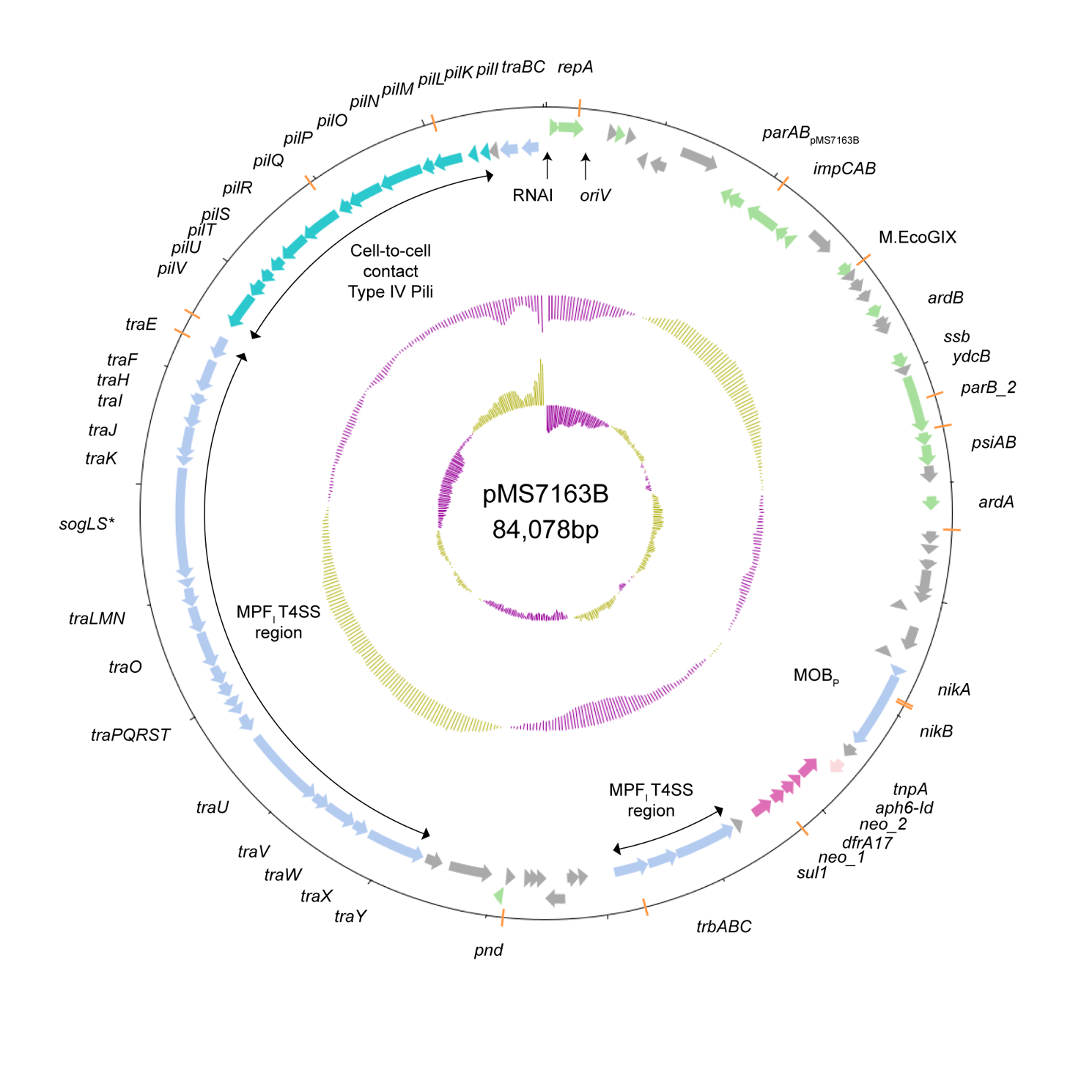
**

**Fig B. Genetic map of pMS7163B.** The rings represent the following from the outermost to the innermost: CDS on the forward strand; CDS on the reverse strand; GC-plot; GC-skew. Arrowheads indicate gene orientation. Plasmid pMS7163B is colour coded based on predicted function: Green – stability/maintenance/replication; Blue – MPF_I_ and conjugation associated; Teal – Type IV pili biogenesis; Dark pink – Resistance; Light pink – Mobile elements; Grey – Hypothetical/Others. Predicted EcoKI restriction sites (AACN_6_GTGC) are shown in orange. The figure was generated using Artemis (18.0.3).


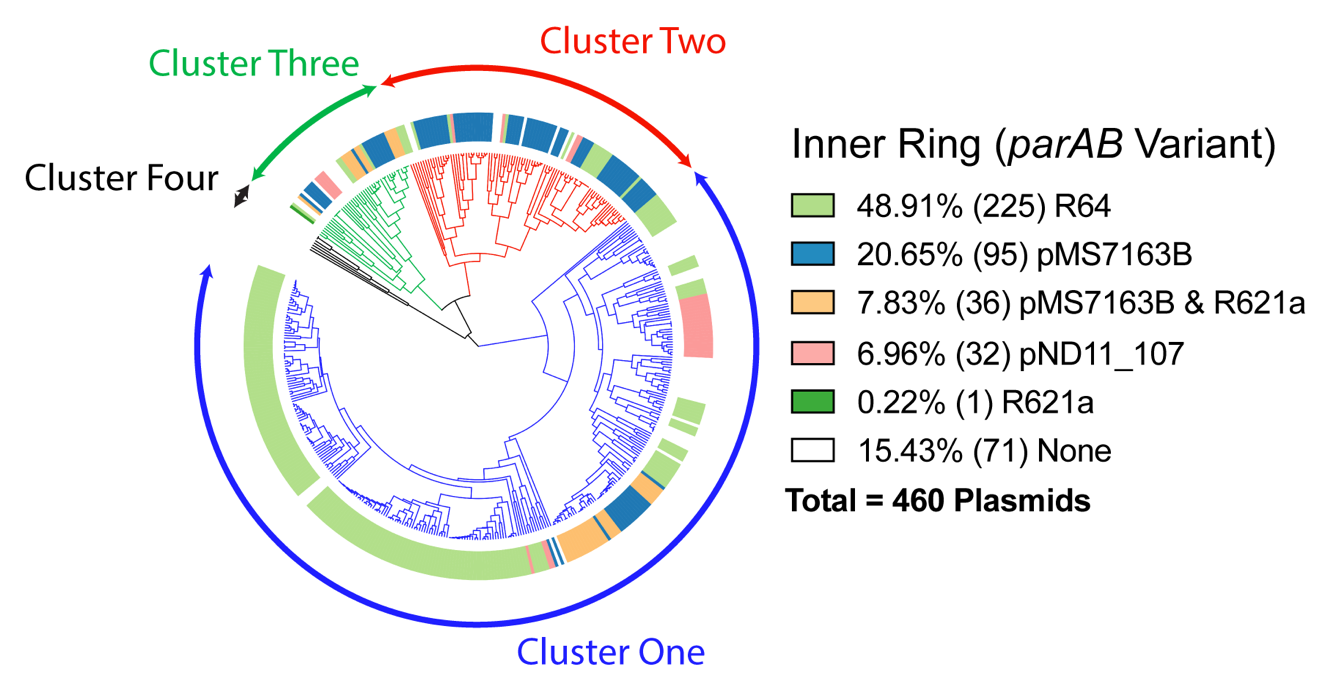


**Fig C. Distribution of *parAB* variants across the 460 I-complex plasmids.** The circular midpoint-rooted cladogram was based on ORF presence/absence using an ORF-based binarized structure network analyses tool and cut into four clusters based on hierarchical clustering and the total within sum of square method. Carriage of *parAB* variants was determined using a BLASTn search at an 80% query length threshold, with the following reference sequences used: pMS7163B (IncB/O backbone with IncZ replicon; CP026855), R64 (IncI1; NC_005014), R621a (IncIɣ; NC_015965), pND11_107 (IncI1; NC_019043).


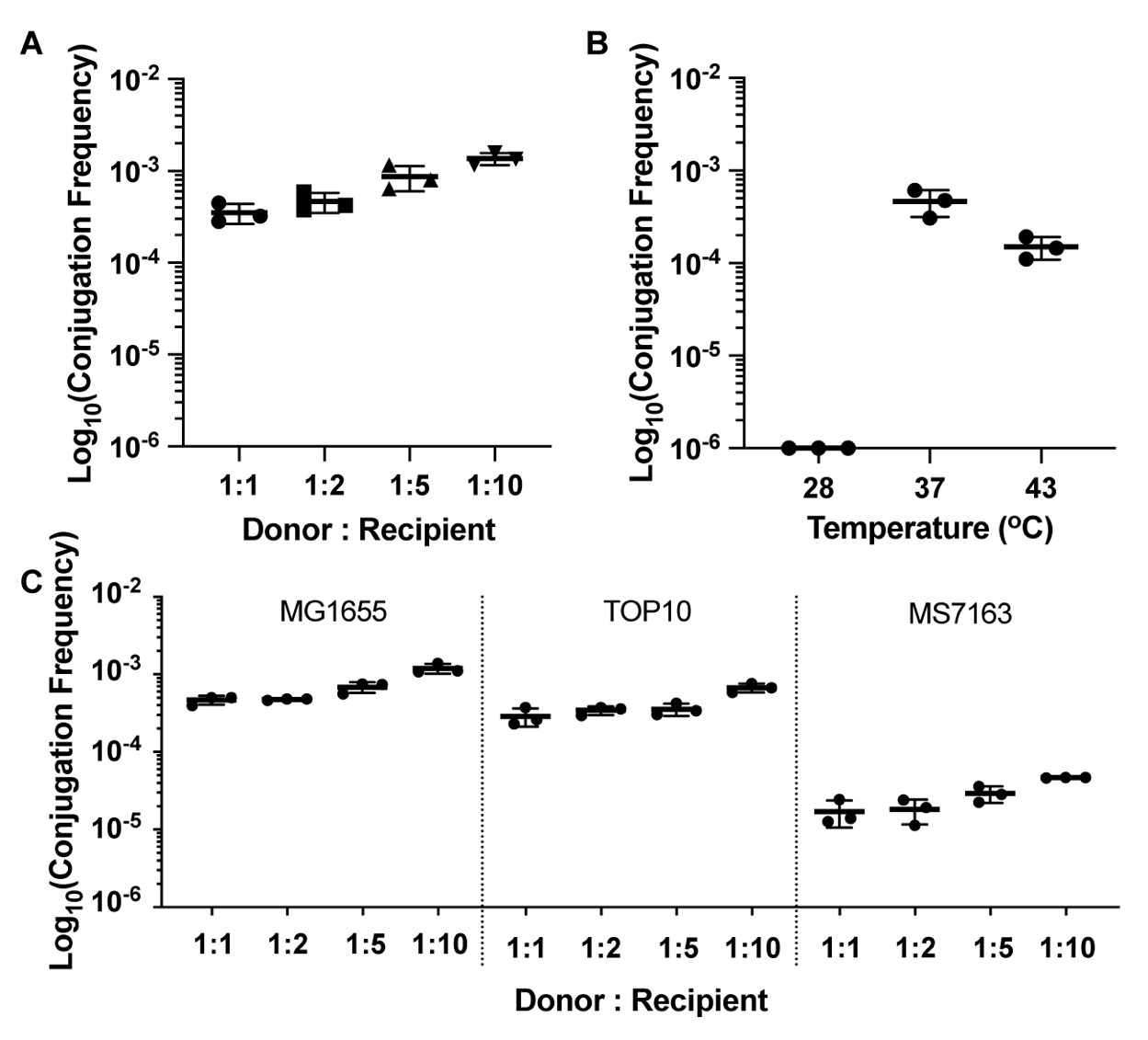


**Fig D. Characterization of pMS7163B surface conjugation frequency. (A) Effect of donor to recipient ratio on pMS7163B conjugation.** Plasmid pMS7163B was conjugated from *E. coli* MG1655 (donor) to *E. coli* J53 (recipient) at 37^o^C for 16 hours. **(B) Effect of temperature on pMS7173B conjugation.** Plasmid pMS7163B was conjugated from *E. coli* MG1655 (donor) to *E. coli* J53 (recipient) at a 1:1 donor to recipient ratio for 16 hours at 28^o^C, 37^o^C, and 43^o^C. **(C) Effect of host strain on pMS7163B conjugation.** Plasmid pMS7163B was conjugated from various *E. coli* donor strains to *E. coli* J53 (recipient) at multiple donor to recipient ratios for 16 hours at 37^o^C. Conjugation frequency for all experiments were calculated as transconjugants/donor. Data represents mean ± SD of three biological replicates.


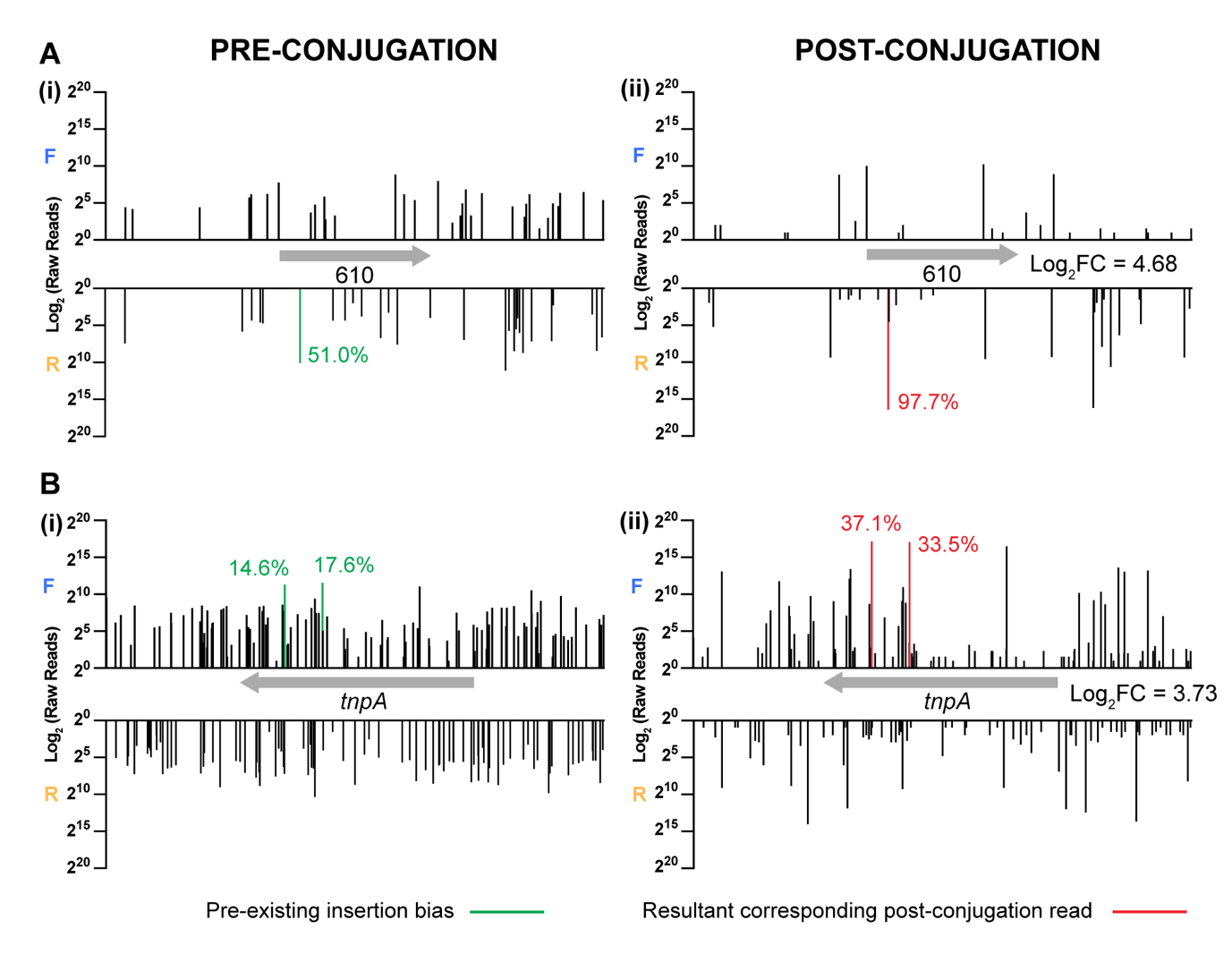


**Fig E. Transposon reads mapped to (A) 610 and (B) *tnpA* in both the (i) pre-conjugation and (ii) post-conjugation libraries.** Log_2_(Raw Reads) on the y-axes represent the number of reads mapped to each mini-Tn*5*-Cm insertion with the promoter orientated in the same direction as the forward strand (top graphs indicated as F), or with the promoter orientated in the direction of the reverse strand (bottom graphs indicated as R) from the first library replicate. Insertion sites that represent >30% reads mapped to their respective coding sequences in the post-conjugation library are shown in red with their proportions in %. The corresponding insertions in the pre-conjugation library are shown in green.


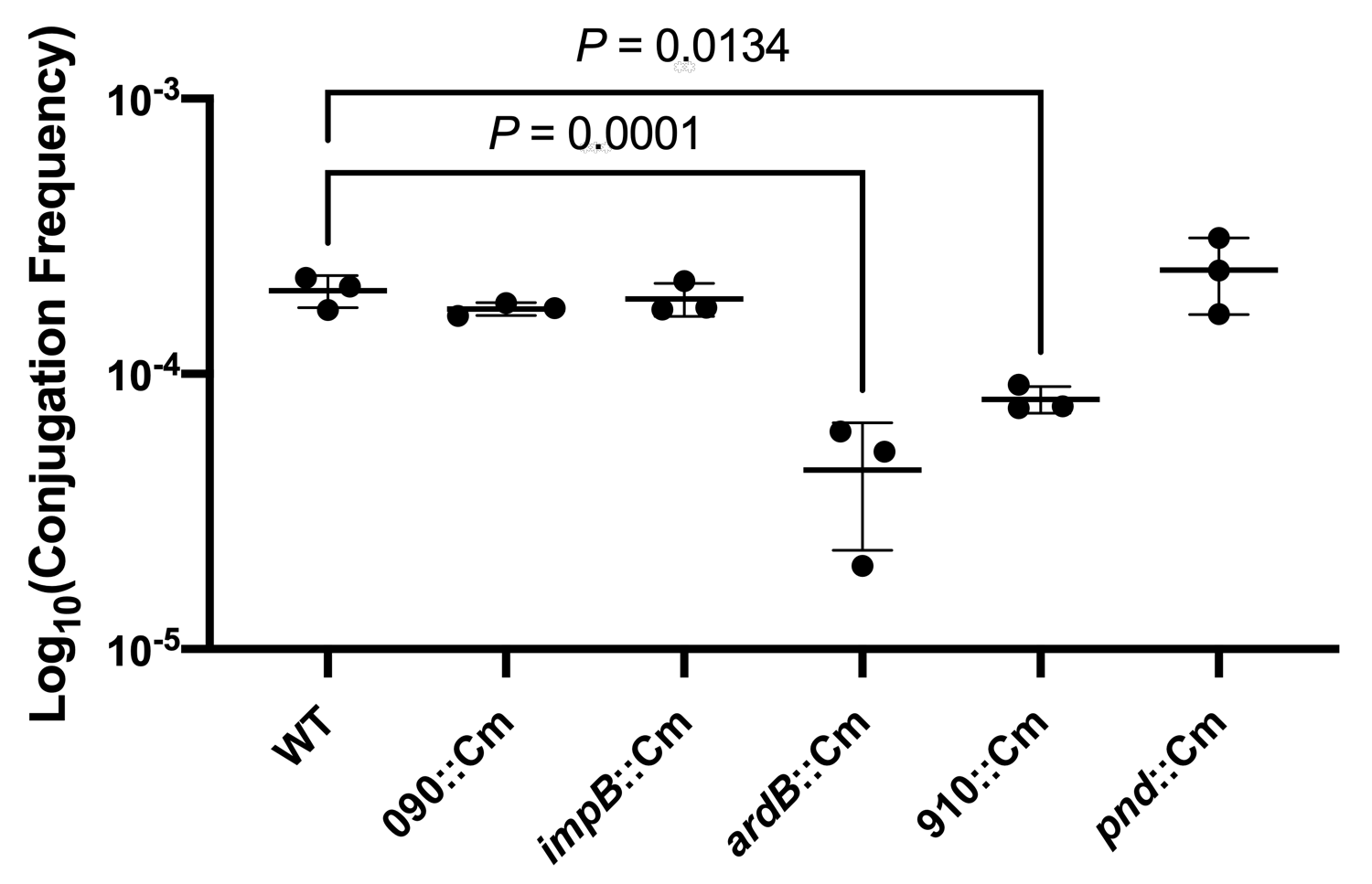


**Fig F. Conjugation frequencies of TOP10 + wildtype pMS7163B, 090::Cm, *impB*::Cm, *ardB*::Cm 910::Cm, *pnd*::Cm**. Conjugation frequency is represented as three biological replicates of Mean ± SD of transconjugants/donor. One-way ANOVA and Sidak’s multiple comparisons were performed on log_10_ transformed values.

**
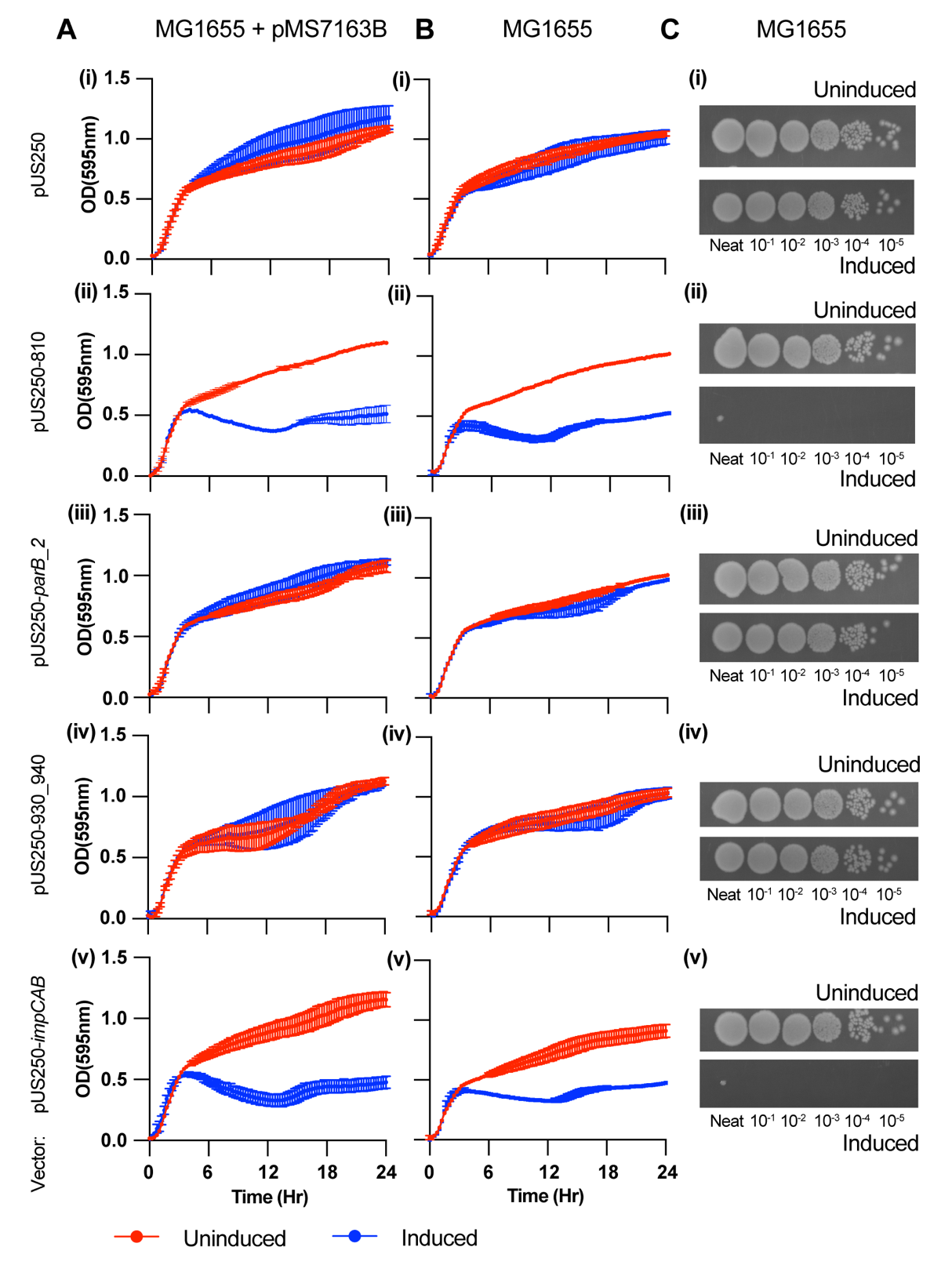
**

**Fig G. (A) Growth curves of MG1655 + pMS7163B and (B) MG1655 carrying the following inducible expression vectors: (i) pUS250 (empty); (ii) p810; (iii) pParB_2; (iv) p930_940; (v) pImpCAB.** Overnight cultures were standardized to OD_600_0.05 and grown in LB + trimethoprim + kanamycin as appropriate. Induction was performed by the addition of cumic acid (100µM). Data represents mean ± SD of three biological replicates. **(C) Serial dilutions of MG1655 carrying the following inducible expression vectors: (i) pUS250 (empty); (ii) p810; (iii) pParB_2; (iv) p930_940; (v) pImpCAB.** Overnight cultures were standardized to OD_600_ 2.0, serially diluted tenfold, and spotted onto LB agar + kanamycin, with and without the presence of cumic acid inducer (100µM). Photos were taken after overnight incubation at 37^o^C and are representative of three biological replicates.

**
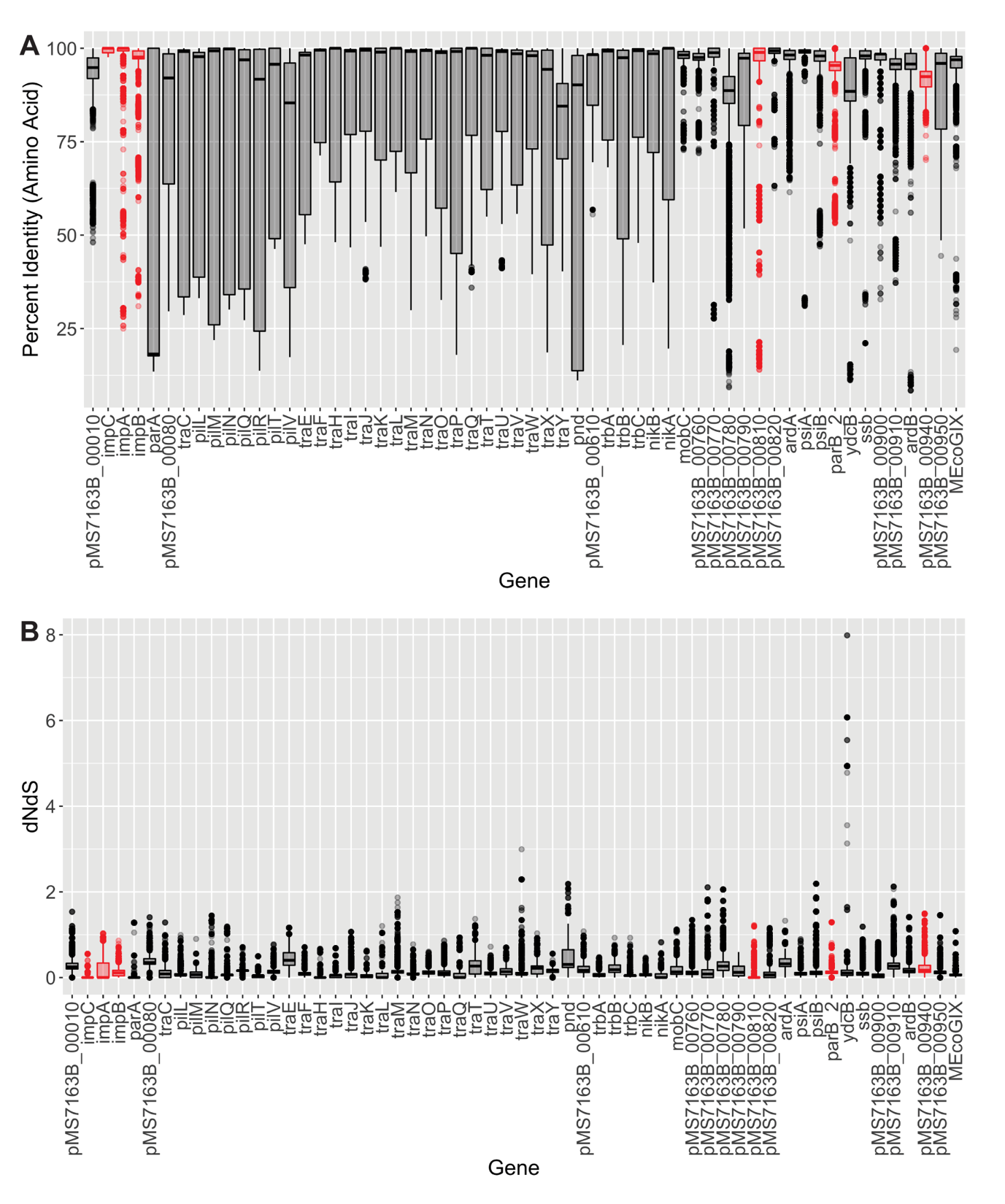
**

**Fig H. Conservation and sequence analyses of broadly conserved genes. (A) Amino acid identity (%) between homologs in the 460 I-complex plasmid database. (B) Ratio of nonsynonymous (dN) to synonymous (dS) substitutions between homologs in the 460 I-complex plasmid database.** Coding sequences from pMS7163B were used as a tBLASTn query against 460 I-complex plasmids using an 80% query length threshold to identify broadly conserved genes. Amino acid identity comparisons were performed using Clustal Omega and dN/dS ratios were estimated using pal2nal and PAML v4.9. Comparisons with dS < 0.01 or > 2 were excluded from analyses due to unreliable dN/dS estimations. Data is represented using Tukey’s boxplot, where the box limits represent first and third quartiles, the internal line represents median, and whiskers represent data within a ±1.5 interquartile range. Dots represent data outside of the whisker range. Genes shown in red are genes that result in decreased host fitness when overexpressed on pMS7163B.

**Fig I. Cycle threshold (CT) values for *repA* gene expression.** Data is representative of 27 biological replicates, and is shown as Mean ± SD.
